# Exploring the effectiveness and safety of stem cell therapy for repair of cartilage defects: a meta-analysis of randomized controlled trials

**DOI:** 10.3389/fcell.2026.1880031

**Published:** 2026-08-07

**Authors:** Mingjing Zhang, Simin Ni, Liqi Ng, Mo Zhou, Jianping Zhang, Nan Tao, Swastina Nath Varma, Aleksandra Ivanova, Rikin Hargunani, Yanwei Du, Chaozong Liu

**Affiliations:** 1 Institute of Orthopaedic and Musculoskeletal Science, Royal National Orthopaedic Hospital, University College London, London, United Kingdom; 2 Royal National Orthopaedic Hospital, London, United Kingdom; 3 Department of Rehabilitation, School of Nursing, Jilin University, Changchun, China

**Keywords:** cartilage defects, clinical outcomes, mesenchymal stem cells, meta-analysis, osteoarthritis, randomized controlled trials, stem cell therapy, subgroup analysis

## Abstract

**Background:**

Osteoarthritis (OA) is one of the most common degenerative joint diseases worldwide, characterized by chronic pain, functional impairment, and progressive cartilage loss. Mesenchymal stem cells (MSCs) therapy has emerged as a promising regenerative strategy due to its chondrogenic and immunomodulatory properties. Although randomized controlled trials (RCTs) have generally demonstrated favorable therapeutic effects, differences in study design, patient characteristics, and treatment protocols have resulted in substantial clinical and methodological heterogeneity. Therefore, an updated meta-analysis is needed to comprehensively evaluate the efficacy and safety of MSC therapy.

**Objective:**

To systematically assess the efficacy and safety of stem cell therapy compared with control interventions in patients with OA with primary emphasis on pain, functional outcomes, cartilage regeneration, and adverse events, and to identify potential factors influencing therapeutic outcomes.

**Methods:**

A comprehensive search of PubMed, Web of Science, Embase, Medline, Scopus, and the Cochrane Library was performed to identify randomized controlled trials (RCTs) published up to 1 August 2025. Clinically relevant outcomes included pain, WOMAC, KOOS subscales, cartilage volume, and adverse events, which were analyzed as key outcome domains reflecting pain, function, structural change, and safety in osteoarthritis. Meta-analysis was conducted using STATA version 15.1 (StataCorp, College Station, TX, United States) to estimate standardized mean differences (SMDs) or odds ratios (ORs) with 95% confidence intervals (CIs). Random-effects models, predefined subgroup analyses, and risk-of-bias assessments were performed.

**Results:**

A total of 24 randomized controlled trials involving 1,389 patients were included. Compared with control interventions, MSC therapy significantly improved pain (SMD = −1.31, 95% CI: −1.82 to −0.81), WOMAC scores (SMD = −0.78, 95% CI: −1.09 to −0.47), cartilage volume (SMD = 0.91, 95% CI: 0.28–1.53), and KOOS subscales. No significant difference was observed in adverse event rates (OR = 1.58, 95% CI: 0.72–3.47). Subgroup analyses suggested that treatment response may vary according to disease etiology, stem cell source, tissue origin, and delivery strategy; however, these findings were exploratory and should be interpreted cautiously because substantial residual heterogeneity remained across several outcomes.

**Conclusion:**

MSC therapy was associated with significant improvements in pain, physical function, cartilage volume, and KOOS outcomes without an apparent increase in adverse events. However, substantial heterogeneity across studies and the exploratory nature of subgroup analyses warrant cautious interpretation of these findings. Further large-scale randomized controlled trials with standardized treatment protocols and long-term follow-up are required to confirm the optimal therapeutic strategy and long-term efficacy and safety of MSC therapy.

## 1 Introduction

Osteoarthritis (OA) is one of the most prevalent degenerative joint disorders worldwide, characterized by progressive degradation of articular cartilage, subchondral bone remodeling, and synovial inflammation. Clinically, OA manifests as joint pain, stiffness, and limited mobility, substantially impairing patients’ quality of life and functional capacity (He et al., 2020). With global population aging, the prevalence of OA continues to increase and has become a leading cause of disability among middle-aged and elderly individuals (Scheuing et al., 2023). According to the Global Burden of Disease (GBD) Study 2021, approximately 595 million individuals were living with symptomatic and radiographically confirmed osteoarthritis in 2020, representing about 7.6% of the global population, highlighting a substantial and growing socioeconomic burden (GBD, 2023).

Current therapeutic strategies for osteoarthritis are generally classified as symptom-modifying or disease-modifying approaches. Most currently available conservative treatments, including nonsteroidal anti-inflammatory drugs (NSAIDs), intra-articular corticosteroids, hyaluronic acid injections, and physical rehabilitation, primarily provide symptom relief by reducing pain and improving joint function; however, they have limited ability to halt or reverse structural cartilage degeneration. In contrast, mesenchymal stem cells (MSCs) therapy has emerged as a promising regenerative strategy with the potential to modify disease progression by promoting cartilage repair, modulating inflammation, and improving the joint microenvironment, although definitive evidence supporting a true disease-modifying effect remains under investigation. Total joint replacement remains the definitive option for end-stage disease but is associated with considerable surgical trauma, high costs, and limited prosthesis longevity (Ambra et al., 2017). Therefore, there remains an unmet clinical need for therapies that can both alleviate symptoms and potentially modify disease progression.

MSCs therapy has emerged as a promising regenerative strategy for osteoarthritis due to its self-renewal capacity, multilineage differentiation potential, and paracrine immunomodulatory effects (Chen et al., 2023). This study focuses exclusively on MSCs, including bone marrow-derived MSCs (BM-MSCs), adipose-derived MSCs (AD-MSCs), and umbilical cord-derived MSCs (UC-MSCs), which represent the most commonly investigated cell sources in randomized controlled trials. Other stem cell types were excluded to ensure clinical and biological homogeneity of the analysis.

Although MSCs possess chondrogenic differentiation potential, current evidence suggests that their therapeutic effects in osteoarthritis are mediated predominantly through paracrine signaling and immunomodulatory mechanisms, including regulation of inflammatory cytokines and improvement of the intra-articular microenvironment. Direct differentiation into chondrocytes is considered to play a less dominant role in cartilage repair. These combined mechanisms may contribute to pain reduction, functional improvement, and cartilage preservation; however, the exact biological pathways remain incompletely understood (Wu et al., 2024).

Clinical studies have reported that intra-articular administration of MSCs may improve pain and function and potentially support cartilage repair (Lopa et al., 2019). However, the current evidence is heterogeneous, and variability in cell source, preparation methods, delivery strategies, and follow-up duration may contribute to inconsistent findings regarding efficacy and safety (Block and Garza, 2017).

Therefore, a comprehensive systematic review and meta-analysis was conducted to synthesize current randomized controlled trial evidence and evaluate the efficacy and safety of MSC therapy compared with control interventions. The primary outcomes of interest included pain relief, functional improvement, structural cartilage outcomes, and treatment-related adverse events. In addition, subgroup analyses were performed to explore potential sources of heterogeneity and factors influencing treatment effects. This study aims to provide an updated evidence-based foundation for clinical decision-making and future research directions.

## 2 Methods

### 2.1 Protocol and registration

This systematic review was conducted in accordance with the Preferred Reporting Items for Systematic Reviews and Meta-Analyses (PRISMA) guidelines (Moher et al., 2009). The protocol was prospectively registered with the International Prospective Register of Systematic Reviews (PROSPERO) under the registration number CRD420251142482.

### 2.2 Search strategy

The search strategy was developed according to the PICO (Population, Intervention, Comparison, Outcome) framework (Huang et al., 2006). A comprehensive literature search was conducted in PubMed, Web of Science, Embase, MEDLINE, Scopus, and the Cochrane Library, with all databases searched on 1 August 2025. Database-specific controlled vocabulary, including Medical Subject Headings (MeSH) for PubMed/MEDLINE and Emtree terms for Embase, was used where applicable in combination with free-text keywords to maximize search sensitivity. No restrictions on publication year were applied. Only studies published in English and designed as randomized controlled trials (RCTs) were considered eligible.

The following search strategy was applied:

(“stem cell” OR “mesenchymal stem cells” OR MSC OR BMSC OR ADSC) AND (“cartilage injury” OR “cartilage defect” OR “knee osteoarthritis” OR KOA) AND (“randomized controlled trial” OR “RCT”)

The objective of this review was to evaluate the efficacy and safety of MSC therapy compared with clinically accepted control interventions for the treatment of knee cartilage defects and osteoarthritis. Therefore, randomized controlled trials employing different clinically relevant comparator interventions (e.g., placebo, hyaluronic acid, microfracture, or other standard treatments) were considered eligible according to the predefined review question, and comparator diversity was taken into account when interpreting the pooled findings.

### 2.3 Inclusion and exclusion criteria

During the initial literature retrieval, automated screening tools, namely the built-in filtering functions provided by each electronic database (e.g., publication type, document type, and study design filters), were applied to exclude review articles, conference abstracts, non-clinical studies, and other clearly ineligible records before manual screening. All records identified through these automated screening tools were subsequently verified manually by two independent reviewers to ensure that no potentially eligible studies were excluded. Two reviewers (Mingjing Zhang and Yanwei Du) then independently screened the titles and abstracts of all remaining records after duplicate removal using EndNote and subsequently assessed the full texts of potentially eligible studies. Any disagreements regarding study eligibility were resolved through discussion with a third reviewer (Chaozong Liu) until consensus was reached. Studies were included if they met the following eligibility criteria.

#### 2.3.1 Inclusion criteria

- Studies reporting at least one clinically relevant outcome, including pain (e.g., VAS), functional outcomes (e.g., WOMAC or KOOS), structural cartilage outcomes (e.g., cartilage volume or MRI-based assessments), or safety outcomes (e.g., adverse events). No minimum follow-up duration was specified as an eligibility criterion; however, follow-up duration was extracted and considered during interpretation of the pooled findings;
- Population: Adult patients (≥18 years) diagnosed with cartilage defects of the knee joint;
- Intervention: Any form of stem cell therapy, including MSCs derived from bone marrow, adipose tissue, or umbilical cord, administered with or without scaffolds (e.g., collagen, hyaluronic acid hydrogel);
- Comparator: Any clinically appropriate control intervention used in randomized controlled trials, including placebo, hyaluronic acid injection, microfracture, autologous chondrocyte implantation, meniscal repair, high tibial osteotomy, knee replacement, or other standard treatment strategies. Owing to the diversity of comparator interventions, this factor was considered during interpretation of the pooled results as a potential source of clinical heterogeneity;
- Outcomes: Studies reporting clinically relevant outcomes including pain, functional scores, structural cartilage outcomes, and safety indicators. These outcomes were analyzed as core evidence domains rather than being assigned hierarchical statistical priority.

#### 2.3.2 Exclusion criteria

- Study design: Randomized crossover trials;
- Population: Participants without cartilage-related pathology;
- Intervention: Non–stem cell–based treatments (e.g., isolated growth factor therapies);
- Comparator: Studies with significant baseline imbalances between intervention and control groups.

### 2.4 Data extraction

Data extraction was conducted independently by two reviewers using a standardized data collection form. The following information was extracted.

- Study characteristics: First author, study design, country of origin, and year of publication;
- Participant characteristics: Age, group allocation, sample size, type and severity of osteoarthritis;
- Stem cell characteristics: Source (autologous vs. allogeneic), and origin (bone marrow, adipose tissue, or umbilical cord);
- Treatment protocol: Delivery method (injection or implantation), treatment frequency, and follow-up duration;
- Outcome measures: WOMAC, VAS, cartilage volume, adverse events, and KOOS subscales—Symptoms (KOOS-S), Activities of Daily Living (KOOS-ADL), and Sports and Recreation (KOOS-SR).

When multiple follow-up assessments were reported within the same randomized controlled trial, outcome data from the longest available follow-up were extracted for quantitative synthesis to avoid double-counting participants and to provide a consistent estimate of the sustained therapeutic effects of MSC therapy.

### 2.5 Risk of bias assessment

The methodological quality of the included studies was independently evaluated by two reviewers using a modified version of the Cochrane risk of bias tool (Min et al., 2020). The following five domains were assessed:

- Randomization process
- Deviations from intended interventions
- Missing outcome data
- Measurement of outcomes
- Selection of reported results

Each domain was rated as having a low risk, some concerns, or a high risk of bias. Any discrepancies between the reviewers were resolved through consultation with a third investigator.

### 2.6 Quality of evidence assessment

To provide a comprehensive evaluation of the available evidence, both the Grading of Recommendations, Assessment, Development and Evaluation (GRADE) framework and the Oxford Centre for Evidence-Based Medicine (OCEBM) Levels of Evidence (2011 version) were applied. These two approaches serve complementary but distinct purposes: the GRADE framework was used to assess the certainty of evidence at the outcome level, while the OCEBM framework was used to classify the level of evidence at the study level based on study design and methodological characteristics.

Specifically, GRADE provides a structured assessment of the certainty of effect estimates across predefined domains (risk of bias, inconsistency, indirectness, imprecision, and publication bias), whereas OCEBM offers a hierarchical classification of evidence strength according to study design. The combined use of these frameworks allows for a more comprehensive evaluation of both outcome-specific certainty and study-level evidence hierarchy, thereby improving the interpretability and robustness of the overall evidence synthesis.

#### 2.6.1 GRADE approach

The overall quality of evidence was evaluated using the Grading of Recommendations, Assessment, Development, and Evaluation (GRADE) framework (Guyatt et al., 2011). Assessment was based on the following five domains:

- Risk of bias
- Inconsistency
- Imprecision
- Indirectness
- Publication bias

#### 2.6.2 GRADE assessment

The certainty of evidence for each outcome was independently assessed by two reviewers according to the GRADE Working Group recommendations. The assessment was conducted at the outcome level across five predefined domains: risk of bias, inconsistency, indirectness, imprecision, and publication bias. Judgments for each domain were made using the predefined GRADE criteria, and any disagreements were resolved through discussion or, when necessary, consultation with a third reviewer.

Statistical heterogeneity (I^2^) was evaluated as one component of the inconsistency domain rather than the sole determinant of evidence certainty. Accordingly, the overall certainty rating was based on an integrated assessment of all five domains and was classified as high, moderate, low, or very low certainty. Decisions to downgrade the certainty of evidence were made when predefined concerns were identified in one or more domains. Downgrading was primarily driven by limitations in risk of bias, substantial unexplained inconsistency, imprecision resulting from limited sample sizes or wide confidence intervals, indirectness when study populations or interventions differed from the review question, and potential publication bias.

#### 2.6.3 Oxford centre for evidence-based medicine (OCEBM)

The level of evidence was further evaluated using the Oxford Centre for Evidence-Based Medicine (OCEBM) Levels of Evidence (2011 version). Evidence levels were assigned according to the study design and methodological characteristics of each included randomized controlled trial. Because the OCEBM framework classifies the strength of evidence at the study level, assessments were performed for individual studies rather than for individual outcomes. The OCEBM framework was used to complement the GRADE approach by providing a study-level classification of evidence, whereas GRADE was applied to assess the certainty of evidence for each outcome. Together, these two frameworks provide complementary perspectives on the overall quality and strength of the available evidence.

### 2.7 Statistical analysis

All statistical analyses were performed using STATA version 15.1 (StataCorp, College Station, TX, United States). For continuous outcomes, standardized mean differences (SMDs) were calculated with Hedges’g, and 95% confidence intervals (CIs) were used to quantify effect sizes.

Between-study heterogeneity was assessed using the Chi-squared (χ^2^) test and the I^2^ statistic. An I^2^ value of ≤50% with p > 0.10 was considered to indicate low heterogeneity, whereas I^2^ > 50% with p < 0.10 was considered to indicate substantial heterogeneity.

Subgroup analyses were conducted to explore potential sources of heterogeneity based on predefined clinical and methodological factors. These analyses were exploratory in nature and involved estimating pooled effects within each subgroup. No formal statistical tests for subgroup interaction were performed; therefore, differences between subgroup estimates should be interpreted descriptively rather than as evidence of statistically significant effect modification.

Publication bias was assessed using funnel plots when at least 10 studies were available for an outcome.

Meta-regression was considered to further explore potential sources of heterogeneity; however, it was not performed due to the limited number of included studies and insufficient reporting of relevant covariates, which would have limited statistical power and reliability.

Sensitivity analyses were not conducted for outcomes with a small number of included studies to avoid unstable or non-informative estimates.

## 3 Results

### 3.1 Literature search results

**FIGURE 1:**
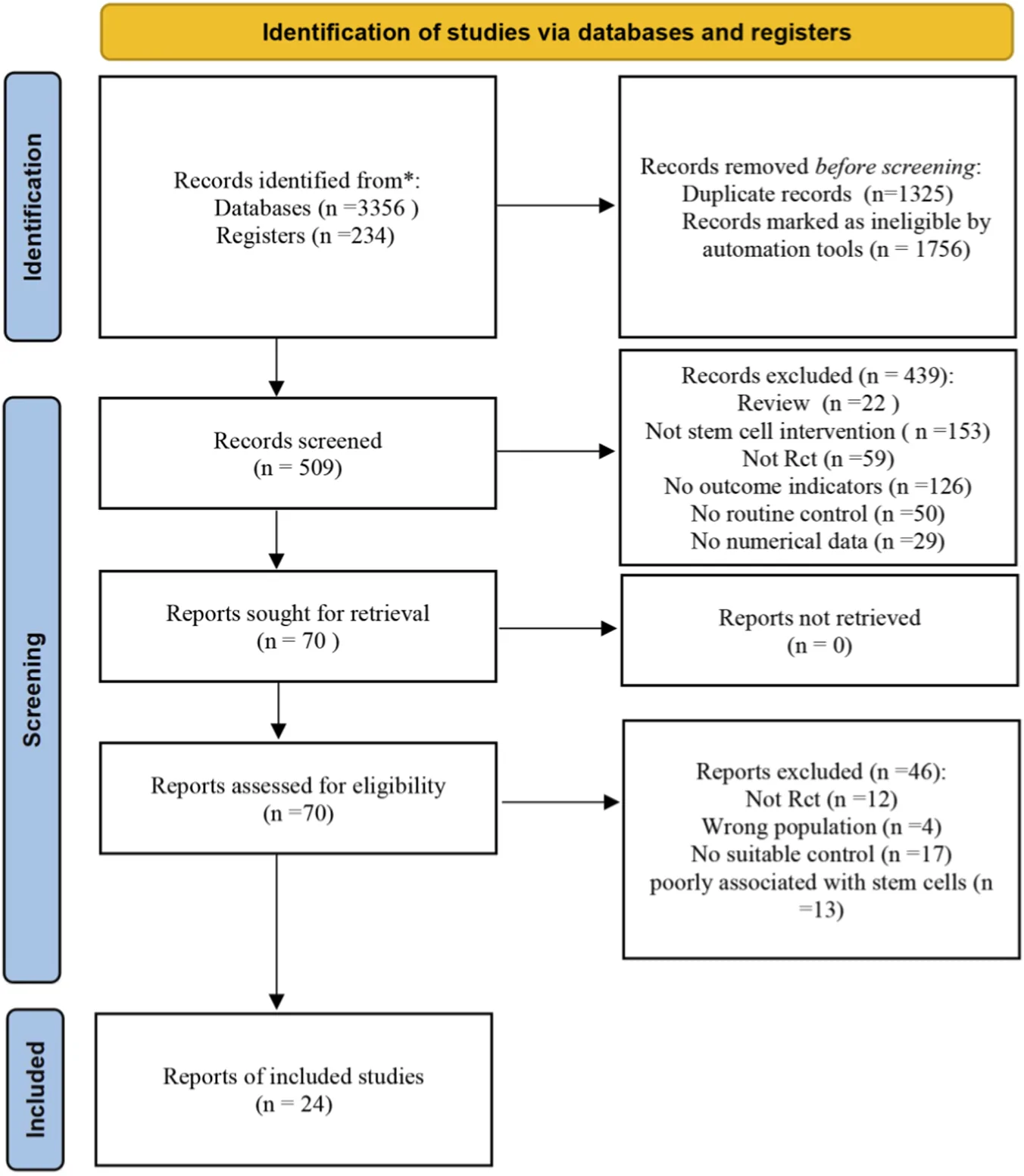
PRISMA flow diagram corresponding to the study selection process.

A total of 3,590 records were identified through database searching. Before title and abstract screening, 1,756 records were removed through automated screening tools (database-specific filtering functions) and duplicate removal during the initial record management process. The remaining records then underwent independent manual title and abstract screening, during which 439 records were excluded because they did not meet the predefined eligibility criteria. Subsequently, 70 full-text articles were assessed for eligibility according to the predefined inclusion and exclusion criteria, of which 46 were excluded after full-text review. Ultimately, 24 randomized controlled trials were included in the meta-analysis (Akgun et al., 2015; Bolandnazar et al., 2024; Garza et al., 2020; Gupta et al., 2016; Hernigou et al., 2018; Hong et al., 2019; Kim et al., 2022; Kim et al., 2023; Lee et al., 2025; Lee et al., 2019; Lu et al., 2019; ZoghAli et al., 2025; Qiao et al., 2020; Sadri et al., 2023; Saw et al., 2013; Saw et al., 2021; Sun et al., 2024; Vangsness et al., 2014; Vega et al., 2015; Wang et al., 2017; Wong et al., 2013; Zhang et al., 2022a; Zhou et al., 2021) (Figure 1).

### 3.2 Study characteristics

This review included 24 randomized controlled trials published between 2015 and 2025, involving a total of 1,389 participants. Among them, 689 patients received stem cell therapy (intervention group), and 700 patients underwent conventional interventions (control group).

The included studies were conducted across 11 countries, including Turkey, Iran, the United States, India, France, South Korea, China, Australia, Spain, Singapore, and Malaysia, representing diverse patient populations. Most participants were adults aged ≥18 years with cartilage defects of the knee joint.

In the intervention groups, MSCs therapy was administered, including bone marrow-derived, adipose-derived, and umbilical cord-derived MSCs, with or without scaffold support (e.g., collagen or hyaluronic acid hydrogel). Control interventions varied substantially across studies and included placebo, hyaluronic acid injection, microfracture, autologous chondrocyte implantation (ACI), meniscal repair, high tibial osteotomy, and knee replacement, reflecting current heterogeneity in clinical practice (Tables 1, 2).

**TABLE 1:** Details of the included studies.

Study	Year	Design	Country	Age		Sample size		Gender		Disease type	Position	Outcome
				I	C	I	C	Male	Female			
Akgun I	2015	RCT	Turkey	32.3 ± 7.9	32.7 ± 10.4	7	7	8	6	Cartilage defect	Knee	①,④,⑥,⑦,⑧,⑨
Bolandnazar NS	2024	RCT	Iran	55.38 ± 6.07	55.38 ± 6.07	26	26	1	28	Osteoarthritis	Knee	①,②,③,④
Garza J.R.	2020	RCT	United States	60.5 ± 7.9	57.1 ± 9.1	13	13	10	16	Osteoarthritis	Knee	②
Garza J.R.-2	2020	RCT	United States	59.5 ± 11.7	57.1 ± 9.1	13	13	13	13	Osteoarthritis	Knee	②
Gupta P.K	2016	RCT	India	58.10 ± 8.23	54.90 ± 8.27	10	10	3	17	Osteoarthritis	Knee	①,②
Gupta P.K-2	2016	RCT	India	57.30 ± 9.45	54.90 ± 8.27	10	10	2	18	Osteoarthritis	Knee	①,②
Gupta P.K-3	2016	RCT	India	55.00 ± 6.72	56.70 ± 5.19	10	10	5	15	Osteoarthritis	Knee	①,②
Gupta P.K-4	2016	RCT	India	54.00 ± 6.73	54.90 ± 8.27	10	10	8	12	Osteoarthritis	Knee	①,②
Hernigou P	2018	RCT	France	18-41	18-41	30	30	12	18	Osteoarthritis	Knee	⑤
Kim J-H	2022	RCT	South Korea	58.3 ± 6.4	59.1 ± 5.9	13	13	7	19	Osteoarthritis	Knee	③,④,⑤
Lee B-W	2025	RCT	South Korea	71.2 ± 5.7	63.8 ± 8.6	11	12	4	20	Osteoarthritis	Knee	①,⑤,⑦
Lee W-S	2019	RCT	South Korea	62.2 ± 6.5	63.2 ± 4.2	12	12	6	18	Osteoarthritis	Knee	①,②,⑤,⑦
Lu L-J	2019	RCT	China	55.03 ± 9.19	59.64 ± 5.97	26	26	6	46	Osteoarthritis	Knee	①,②,④,⑤
Sadri B	2023	RCT	Iran	52.8 ± 7.5	56.1 ± 7.2	18	18	4	36	Osteoarthritis	Knee	①,②,④,⑤,⑥,⑦,⑧,⑨
Sun H	2024	RCT	China	54.57 ± 7.79	54.55 ± 7.18	23	23	18	27	Osteoarthritis	Knee	②,⑤
Wang Y	2017	RCT	Australia	26.0 ± 3.6	26.9 ± 10.3	11	6	10	5	ACL reconstruction	Knee	④,⑪
Zhang S	2022	RCT	China	53.98 ± 13.69	55.63 ± 12.18	51	64	30	96	Osteoarthritis	Knee	①,②,④,⑤
Zhang Y	2022	RCT	China	50.83 ± 10.88	52.87 ± 9.35	47	48	38	57	Osteoarthritis	Knee	①
Zhou Y	2021	RCT	China	52.27 ± 1.17	51.77 ± 7.55	30	30	13	47	Osteoarthritis	Knee	①,②,③
Masoumeh Z	2025	RCT	Iran	57.9 ± 6.56	60.55 ± 7.51	30	27	9	48	Osteoarthritis	Knee	①,②
Hong Z	2018	RCT	China	53 ± 10.97	53 ± 10.97	16	16	3	13	Osteoarthritis	Knee	①,③
Kim K	2023	RCT	South Korea	63.7 ± 7.1	63.8 ± 7.1	125	127	65	187	Osteoarthritis	Knee	①,②,⑥,⑦,⑧,⑨,⑩
Qiao Z	2020	RCT	China	62.0 ± 8.33	59.7 ± 7.12	10	10	12	8	Osteoarthritis	Knee	②,⑤
Thomas C	2014	RCT	United States	46	46	18	18	NA	NA	Cartilage defect	Knee	⑤
Thomas C-2	2014	RCT	United States	46	46	18	18	NA	NA	Cartilage defect	Knee	⑤
Vega A	2015	RCT	Spain	57 ± 9	57 ± 9	15	15	13	17	Osteoarthritis	Knee	①,②,⑤
Wong KL	2013	RCT	Singapore	36-54	24-54	28	28	27	29	Cartilage defect	Knee	③
Saw Ky	2013	RCT	Malaysia	22-48	22-45	25	24	17	32	Cartilage defect	Knee	⑤,⑩
Saw Ky	2021	RCT	Malaysia	22-55	22-55	33	36	17	16	Cartilage defect	Knee	③,⑥,⑦,⑧,⑨,⑩,⑫

RCT:Randomized Controlled Trial,I:intervention group, C:control group, ①:VAS(Visual Analog Scale), ②:WOMAC-T (Western Ontario and McMaster Universities Osteoarthritis Index - Total), ③:cartilage score, ④:cartilage volume, ⑤:complication, ⑥:Koos-s (Knee injury and Osteoarthritis Outcome Score-symptoms), ⑦Koos-ADL (Knee injury and Osteoarthritis Outcome Score - Function in Daily Living), ⑧Koos-SR (Knee injury and Osteoarthritis Outcome Score - Sport/Recreation), ⑨Koos-Qol (Knee injury and Osteoarthritis Outcome Score - Quality of Life), ⑩IKDC(International Knee Documentation Committee Subjective Knee Form), ⑪Koos-p (Knee injury and Osteoarthritis Outcome Score-pain).

**TABLE 2:** Intervention details of the included studies.

Study	Year	Severity of disease		Stem cell source		I		C	Follow-up time
		ICRS	Kellgren-lawrence	Source	Origian	Implantation	Scallford	Method	
Akgun I	2015	III-IV	​	Autologous	Bone synovial tissue	Implant	Type I/III collagen membrane	Autologous chondrocyte transplantation	2Y
Bolandnazar NS	2024	​	II-III	Autologous	Placenta	Injection	No	Placebo	0.5Y
Garza J.R.	2020	​	III-IV	Autologous	Adipose	Injection	No	Placebo	1Y
Garza J.R.-2	2020	​	III-IV	Autologous	Adipose	Injection	No	Placebo	1Y
Gupta P.K	2016	​	II-III	Allogeneic	Bone marrow	Injection	No	Placebo	1Y
Gupta P.K-2	2016	​	II-III	Allogeneic	Bone marrow	Injection	No	Placebo	1Y
Gupta P.K-3	2016	​	II-III	Allogeneic	Bone marrow	Injection	No	Placebo	1Y
Gupta P.K-4	2016	​	II-III	Allogeneic	Bone marrow	Injection	No	Placebo	1Y
Hernigou P	2018	​	IV	Allogeneic	Bone marrow	Injection	No	Knee replacement	12Y
Kim J-H	2022	​	II-IV	Autologous	Adipose	Injection	No	High tibial osteotomy	2Y
Lee B-W	2025	​	I-IV	Allogeneic	Bone marrow	Injection	No	Placebo	1Y
Lee W-S	2019	​	II-IV	Autologous	Adipose	Injection	No	Placebo	0.5Y
Lu L-J	2019	​	I-III	Autologous	Adipose	Injection	No	Injection of hyaluronic acid	1Y
Sadri B	2023	​	II-III	Allogeneic	Adipose	Injection	No	Placebo	1Y
Sun H	2024	​	I-III	Autologous	Adipose	Injection	No	High tibial osteotomy	2Y
Wang Y	2017	​	<I	Allogeneic	Bone marrow	Injection	No	Injection of hyaluronic acid	2Y
Zhang S	2022	​	II-III	Autologous	Adipose	Injection	No	Injection of hyaluronic acid	5Y
Zhang Y	2022	​	II-III	Autologous	Bone marrow	Injection	No	Injection of hyaluronic acid	1Y
Zhou Y	2021	​	≤III	Autologous	Adipose	Injection	No	Meniscal repair	1Y
Masoumeh Z	2025	​	II-III	Allogeneic	Adipose	Injection	No	Injection of triamcinolone acetonide	2Y
Hong Z	2018	​	II-III	Autologous	Adipose	Injection	No	Injection of hyaluronic acid	1Y
Kim K	2023	​	III	Autologous	Adipose	Injection	No	Placebo	0.5Y
Qiao Z	2020	​	III	Autologous	Adipose	Injection	No	Injection of hyaluronic acid	2Y
Thomas C	2014	​	​	Allogeneic	Bone marrow	Injection	No	Placebo	2Y
Thomas C-2	2014	​	​	Allogeneic	Bone marrow	Injection	No	Placebo	2Y
Vega A	2015	​	II-IV	Allogeneic	Bone marrow	Injection	No	Injection of hyaluronic acid	1Y
Wong KL	2013	IV	​	Autologous	Bone marrow	Injection	No	High tibial osteotomy + injection of hyaluronic acid	2Y
Saw Ky	2013	III-IV	​	Autologous	Peripheral blood	Injection	No	Injection of hyaluronic acid	2Y
Saw Ky	2021	III-IV	​	Autologous	Peripheral blood	Injection	No	Injection of hyaluronic acid	Follow-up time

I, intervention group; C, control group.

The duration of follow-up varied widely across studies, ranging from 6 months to 12 years. Outcomes were therefore synthesized across heterogeneous follow-up timepoints, and this temporal variability should be considered when interpreting the pooled effect estimates.

### 3.3 Risk of bias assessment

**FIGURE 2:**
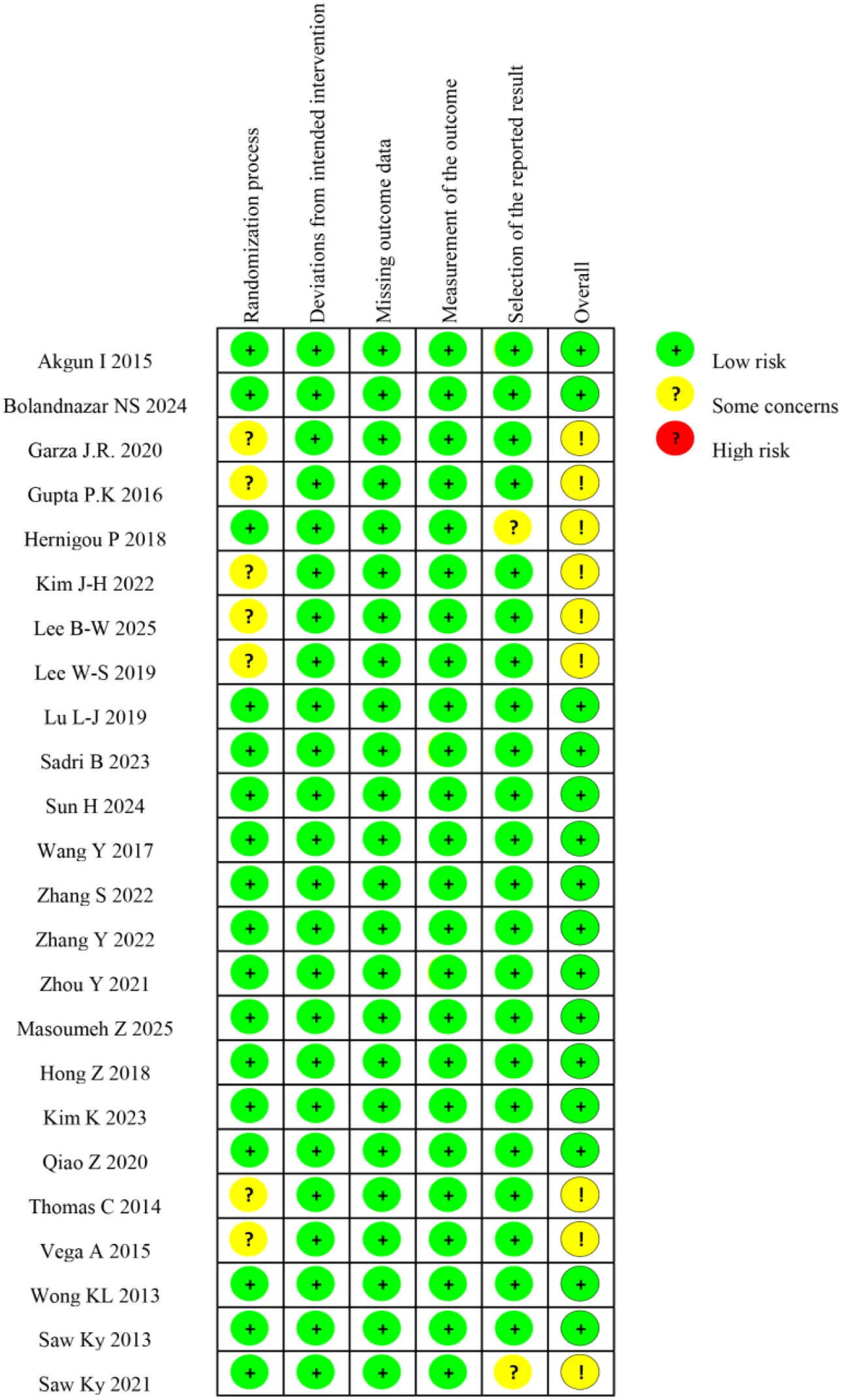
Risk of bias assessment summary of included randomized controlled trials. The figure presents domain-level judgments (randomization process, deviations from intended interventions, missing outcome data, measurement of outcomes, and selection of reported results), highlighting variability across studies rather than a uniform overall risk classification.

All 24 included studies reported their randomization methods, with 17 providing detailed descriptions of both sequence generation and allocation concealment. Among these studies, 2 employed triple blinding, 14 were double-blinded, 6 were single-blinded, and 2 did not clearly report their blinding procedures. Across the included randomized controlled trials, risk of bias varied across methodological domains. Allocation concealment and blinding procedures were the most common sources of concern, whereas outcome measurement and incomplete outcome data were generally assessed as low risk. Overall, most studies were rated as either low risk or having some concerns in at least one domain (Figure 2; Supplementary Table 1).

Risk of bias was assessed at both the domain and study levels using the Cochrane Risk of Bias 2.0 tool. Therefore, no single overall summary judgment was assigned across all studies. Interpretation of risk of bias should instead be based on the distribution of domain-level assessments, which indicated generally low risk in several key methodological domains, with residual concerns primarily related to blinding and allocation concealment.

### 3.4 Meta-analysis results

#### 3.4.1 Pain

**FIGURE 3:**
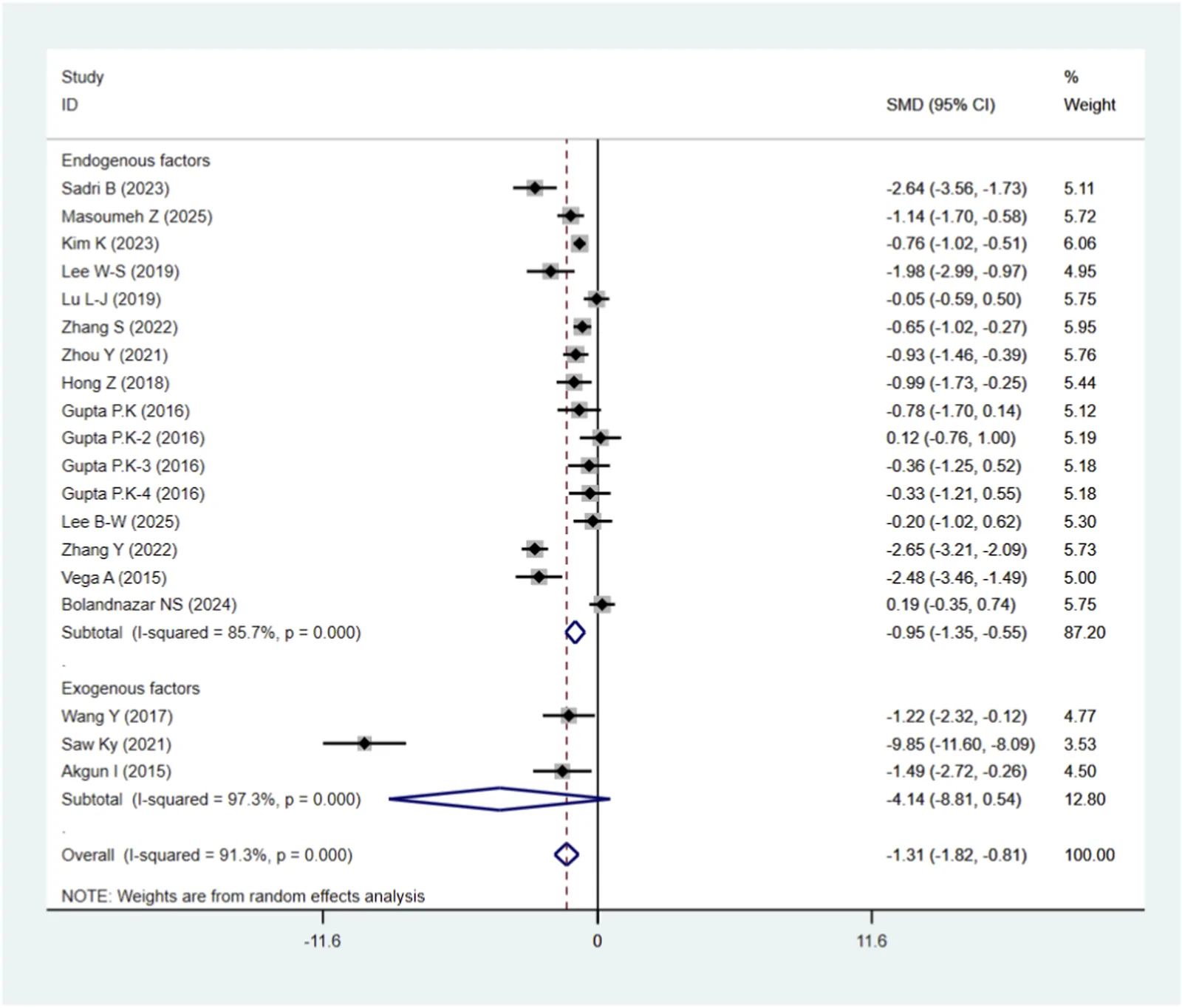
Forest plot of Pain.

Using a random-effects model, stem cell therapy was associated with a significant reduction in pain compared with control interventions (standardized mean difference [SMD] = −1.31; 95% confidence interval [CI]: 1.82 to −0.81). However, substantial heterogeneity was observed among the studies (I^2^ = 91.3%, p < 0.001) (Figure 3).

Subgroup analyses were performed according to predefined clinical characteristics. The idiopathic osteoarthritis subgroup included 22 studies (n = 1114 participants), while the trauma-induced osteoarthritis subgroup included 7 studies (n = 227 participants). Although subgroup analyses suggested potential differences in treatment effects across etiological categories, these findings should be interpreted as exploratory given the substantial residual heterogeneity (Supplementary Table 2).

Specifically, a larger effect size was observed in idiopathic osteoarthritis (SMD = −0.95; 95% CI: 1.35 to −0.55), whereas a comparatively larger and less precise estimate was observed in trauma-induced osteoarthritis (SMD = −4.14; 95% CI: 8.81 to 0.54), likely reflecting the limited number of studies and potential small-study effects.

Regarding stem cell source, autologous stem cell therapy showed greater effect estimates compared with allogeneic stem cells (autologous: SMD = −1.88; 95% CI: 2.82 to −0.95; allogeneic: SMD = −0.80; 95% CI: 1.23 to −0.38). The autologous subgroup included 10 studies (n = 543 participants), and the allogeneic subgroup included 9 studies (n = 465 participants).

The funnel plot did not show clear evidence of publication bias (Supplementary Figure 1). However, interpretation of funnel plots should be made with caution due to the relatively small number of included studies and substantial between-study heterogeneity, which may limit the statistical power to detect asymmetry.

#### 3.4.2 WOMAC total Score

A reduction in the WOMAC total score after treatment reflects improved clinical outcomes and therapeutic efficacy.

**FIGURE 4:**
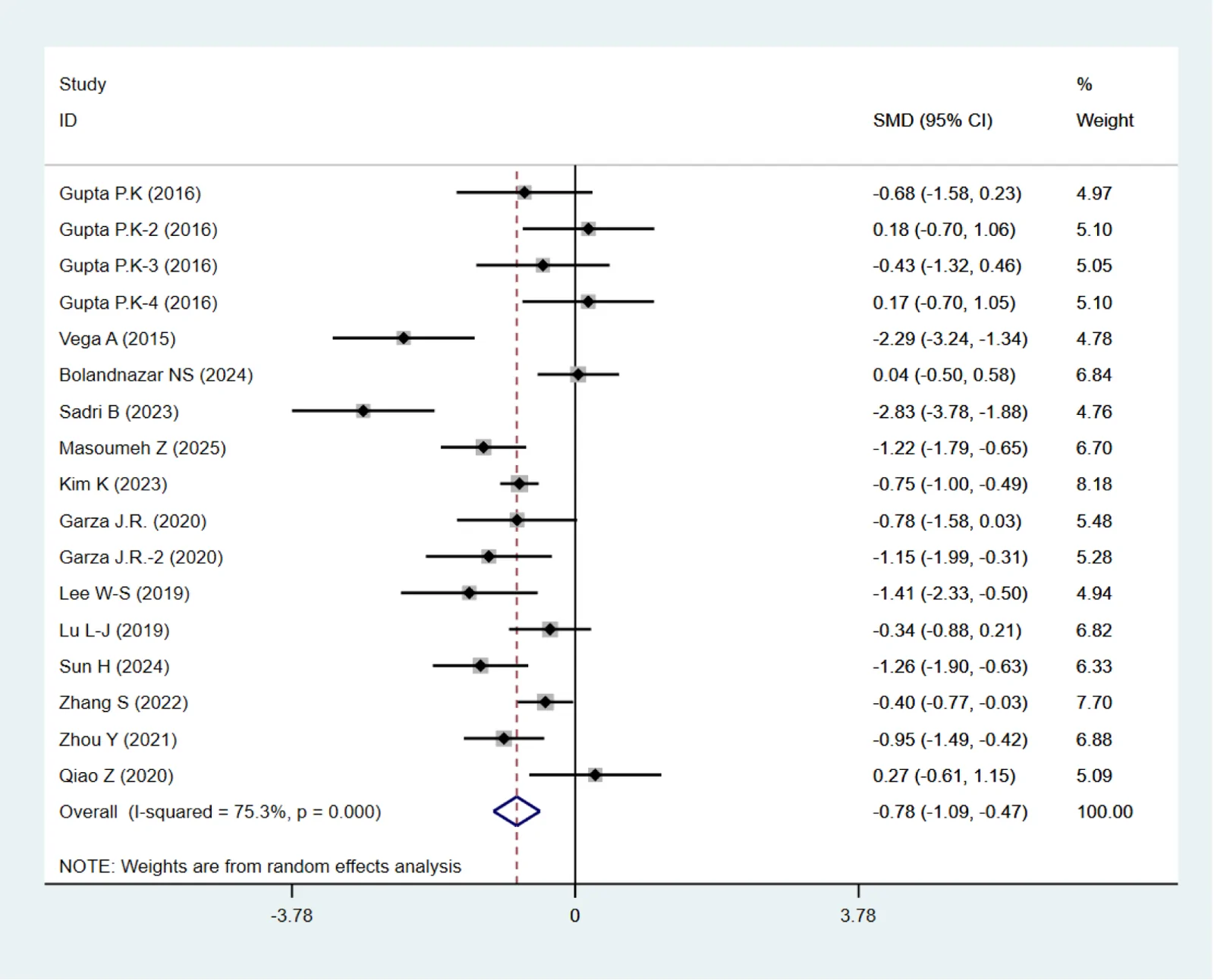
Forest plot of WOMAC.

Using a random-effects model, stem cell therapy was associated with a significant decrease in WOMAC total scores compared with control interventions (standardized mean difference [SMD] = −0.78; 95% confidence interval [CI]: 1.09 to −0.47), although considerable heterogeneity was detected (I^2^ = 75.3%, p < 0.001) (Figure 4).

Although subgroup analyses were performed according to stem cell source and tissue origin, substantial residual heterogeneity remained, indicating that additional clinical and methodological factors likely contributed to the observed variability (Supplementary Table 3). The stem cell source subgroup included 17 studies (n = 876 participants), and the tissue origin subgroup included 16 studies (n = 824 participants).

The funnel plot showed no evidence of publication bias (Supplementary Figure 2). However, funnel plot interpretation should be cautious due to the limited number of included studies and the presence of substantial heterogeneity, which may reduce the statistical power to detect publication bias.

#### 3.4.3 Cartilage Volume

An increase in cartilage volume following treatment reflects structural improvement and therapeutic efficacy.

**FIGURE 5:**
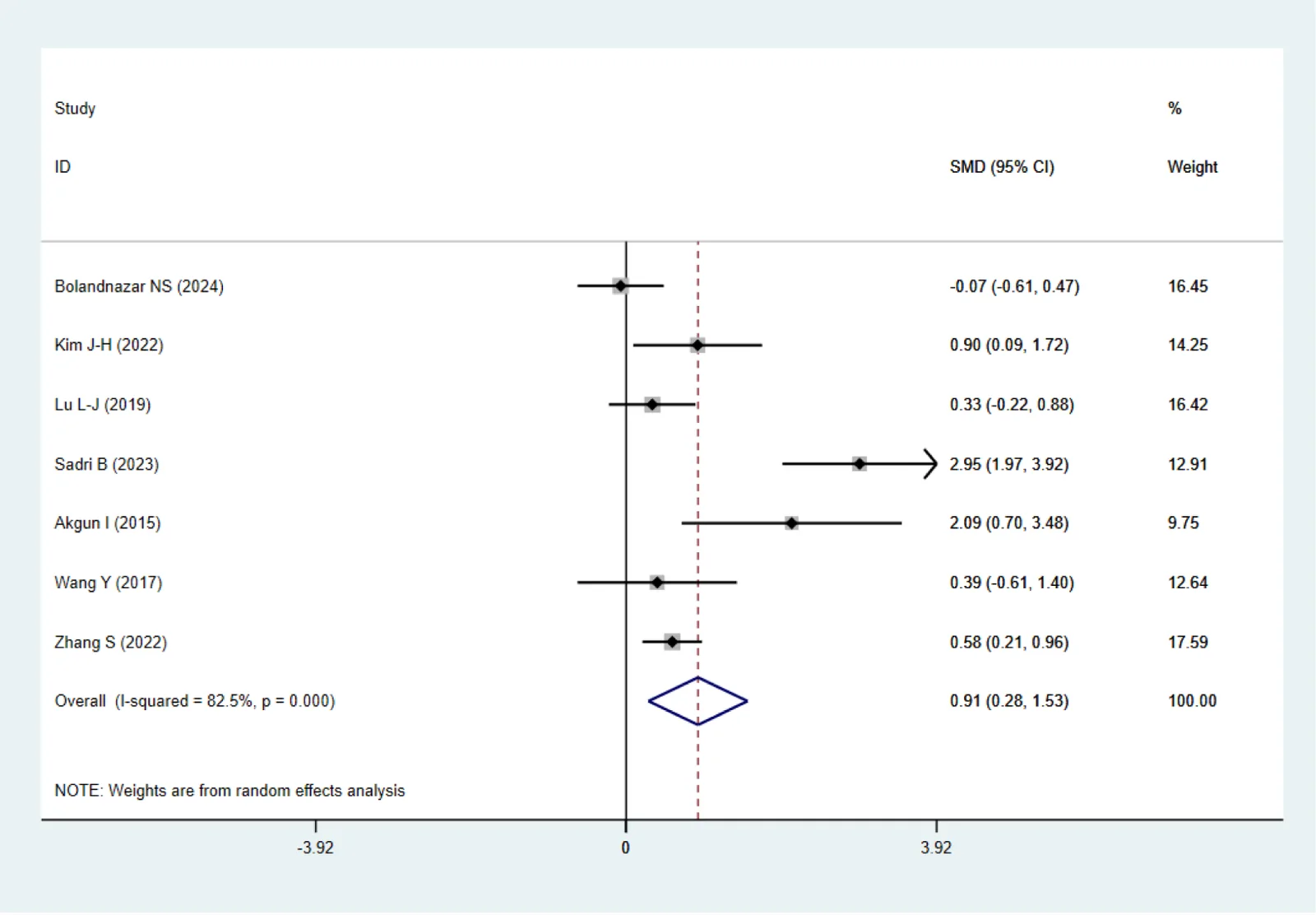
Forest plot of Cartilage volume.

Using a random-effects model, stem cell therapy was associated with a significant increase in cartilage volume compared with control interventions (standardized mean difference [SMD] = 0.91; 95% confidence interval [CI]: 0.28–1.53), despite considerable heterogeneity (I^2^ = 82.5%, p < 0.001) (Figure 5).

Subgroup analyses indicated that the therapeutic effect of stem cell therapy was independent of disease etiology. The disease etiology subgroup included 7 studies (n = 312 participants). Moreover, variations in stem cell source, harvesting site, or scaffold application did not fully explain the substantial between-study heterogeneity, suggesting that additional clinical and methodological factors remained unaccounted for the observed heterogeneity (Supplementary Table 4).

Specifically, bone marrow-derived stem cells demonstrated superior efficacy compared to adipose-derived stem cells (bone marrow: SMD = 2.09; 95% CI: 0.70 to 3.48; adipose: SMD = 1.00; 95% CI: 0.24–1.96). The bone marrow-derived subgroup included 4 studies (n = 229 participants), and the adipose-derived subgroup included 1 studies (n = 17 participants). In subgroup analyses, studies without scaffold-assisted delivery demonstrated comparatively larger pooled effect estimates than studies using scaffold-assisted implantation. However, these subgroup analyses were exploratory and should not be interpreted as direct comparisons between treatment strategies (without scaffolds: SMD = 0.78; 95% CI: 0.14 to 1.41; with scaffolds: SMD = −1.49; 95% CI: 2.72 to −0.26). The non-scaffold subgroup included 6 studies (n = 298 participants), while the scaffold-assisted subgroup included 1 studies (n = 14 participants).

The funnel plot revealed no evidence of publication bias (Supplementary Figure 3). However, funnel plot interpretation should be cautious due to the limited number of included studies and the presence of substantial heterogeneity, which may reduce the statistical power to detect publication bias.

#### 3.4.4 Adverse Events

**FIGURE 6:**
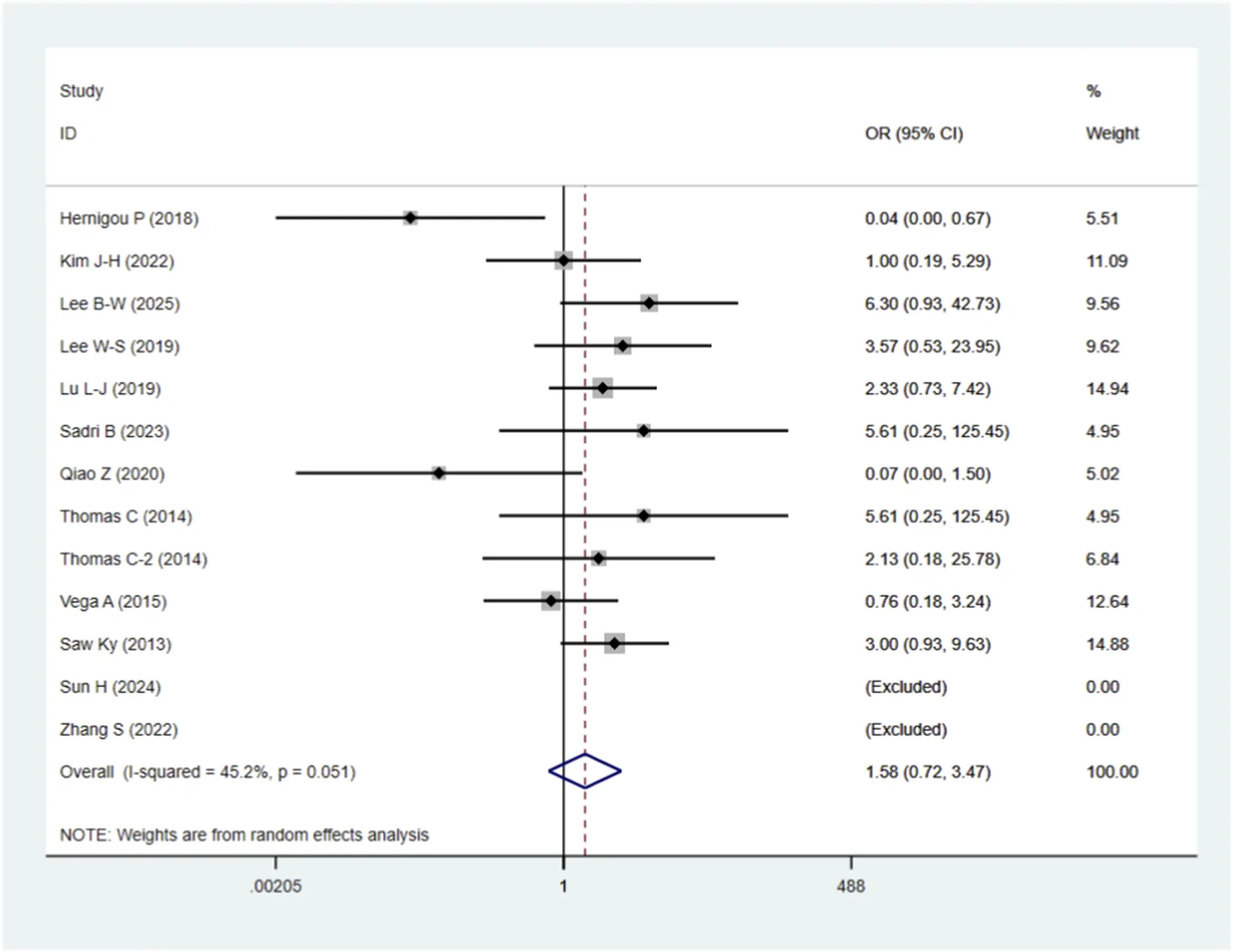
Forest plot of adverse events.

A random-effects model showed no significant difference in adverse event incidence between stem cell therapy and control interventions (odds ratio [OR] = 1.58; 95% confidence interval [CI]: 0.72–3.47). The degree of heterogeneity was moderate (I^2^ = 45.2%, p **=** 0.051)) (Figure 6).

Funnel plot analysis revealed no apparent asymmetry, suggesting a low likelihood of publication bias (Supplementary Figure 4). However, funnel plot interpretation should be cautious due to the limited number of included studies and the presence of substantial heterogeneity, which may reduce the statistical power to detect publication bias.

#### 3.4.5 KOOS-S (Symptoms)

Higher KOOS-S (Symptoms) scores following treatment generally indicate improved symptom relief and therapeutic efficacy.

In the random-effects model, stem cell therapy was associated with a statistically significant improvement in KOOS-S (Symptoms) scores compared with control interventions (standardized mean difference [SMD] = 3.04; 95% confidence interval [CI]: 0.11–5.96). However, considerable heterogeneity was detected (I^2^ = 97.7%, p < 0.001) (Supplementary Figure 5).

Subgroup analyses indicated that the therapeutic effect of stem cell therapy was independent of the underlying etiology. The KOOS-S subgroup analysis included 4 studies (n = 371 participants). Additionally, variations in stem cell source, harvesting site, or scaffold application explained only a small proportion of the observed heterogeneity, while substantial residual heterogeneity remained (Supplementary Table 5).

The funnel plot indicated a potential risk of publication bias (Supplementary Figure 6). However, funnel plot interpretation should be cautious due to the limited number of included studies and the presence of substantial heterogeneity, which may reduce the statistical power to detect publication bias.

#### 3.4.6 KOOS-ADL (activities of daily Living)

Higher KOOS-ADL (Activities of Daily Living) scores reflect better functional performance and clinical improvement.

Using a random-effects model, stem cell therapy was associated with significantly greater improvements in KOOS-ADL scores compared with control interventions (standardized mean difference [SMD] = 2.49; 95% confidence interval [CI]: 0.63–4.36). However, considerable heterogeneity was detected (I^2^ = 96.7%, p < 0.001) (Supplementary Figure 7).

Subgroup analyses showed that the treatment effect was independent of the underlying etiology. The KOOS-ADL subgroup included four studies (n = 418 participants). Other variables, including stem cell source, extraction site, and the use of scaffolds, explained only a small proportion of the observed heterogeneity, while substantial residual heterogeneity remained (Supplementary Table 6).

Notably, stem cell therapies using autologous cells showed greater efficacy than those using allogeneic sources (autologous: SMD = 3.70; 95% CI: 0.17 to 7.24; allogeneic: SMD = 1.30; 95% CI: 0.60–3.20). The autologous subgroup included three studies (n = 107 participants), and the allogeneic subgroup included three studies (n = 311participants). Similarly, better outcomes were reported in studies that did not use scaffolds compared to those that did (without scaffolds: SMD = 2.68; 95% CI: 0.50 to 4.85; with scaffolds: SMD = 1.60; 95% CI: 0.34–2.86). The non-scaffold subgroup included 1 studies (n = 14 participants), while the scaffold-assisted subgroup included 5 studies (n = 404 participants).

The funnel plot indicated a potential presence of publication bias (Supplementary Figure 8). However, funnel plot interpretation should be cautious due to the limited number of included studies and the presence of substantial heterogeneity, which may reduce the statistical power to detect publication bias.

#### 3.4.7 KOOS-SR (Sports and Recreation)

Higher KOOS-SR (Sports and Recreation) scores after treatment generally indicate better therapeutic outcomes.

In the random-effects model, stem cell therapy was associated with a significant improvement in KOOS-SR scores compared with control interventions (standardized mean difference [SMD] = 2.39; 95% confidence interval [CI]: 0.26–4.51), although substantial heterogeneity was detected (I^2^ = 96.7%, p < 0.001) (Supplementary Figure 9).

Subgroup analyses indicated that the treatment effect was independent of the underlying etiology. The KOOS-SR subgroup included four studies (n = 371 participants). Variations in stem cell source, harvesting site, or scaffold application explained only a small proportion of the observed heterogeneity, while substantial residual heterogeneity remained (Supplementary Table 7).

Notably, autologous stem cell therapy demonstrated greater efficacy than allogeneic approaches (autologous: SMD = 2.82; 95% CI: 0.08 to 5.55; allogeneic: SMD = 1.98; 95% CI: 1.09–5.04). The autologous subgroup included two studies (n = 83 participants), and the allogeneic subgroup included two studies (n = 288 participants).

The funnel plot revealed a potential presence of publication bias (Supplementary Figure 10). However, funnel plot interpretation should be cautious due to the limited number of included studies and the presence of substantial heterogeneity, which may reduce the statistical power to detect publication bias.

### 3.5 Quality of evidence

#### 3.5.1 GRADE assessment of certainty

The certainty of evidence for each outcome was assessed using the GRADE framework. Overall, the included outcomes were rated as high, moderate, or low certainty (Supplementary Table 8). The primary reasons for downgrading certainty were risk of bias and inconsistency across studies, with additional downgrading in some outcomes due to imprecision and potential publication bias.

In particular, outcomes demonstrating substantial unexplained heterogeneity were downgraded under the inconsistency domain where appropriate. Detailed justifications for each GRADE judgment are provided in Supplementary Table 8.

This meta-analysis synthesized multiple clinically relevant outcome domains (pain, function, structural outcomes, and safety) rather than testing a single primary hypothesis. Therefore, no formal adjustment for multiple comparisons was applied, consistent with methodological guidance for systematic reviews focused on effect estimation across predefined outcomes.

#### 3.5.2 Oxford centre for evidence-based medicine (OCEBM) levels of evidence

According to the Oxford Centre for Evidence-Based Medicine (OCEBM) 2011 guidelines, the included randomized controlled trials for therapeutic interventions, diagnostic performance, prognostic outcomes, and safety evaluation were classified as Level 1 evidence. Evidence related to prevalence estimates was classified as Level 2, reflecting differences in study design and methodological context (Supplementary Table 9).

## 4 Discussion

This meta-analysis of 24 randomized controlled trials including 1,389 patients suggests that MSCs therapy is associated with improvements in pain, joint function, and cartilage volume in patients with osteoarthritis (OA), without an observed increase in adverse events. The present study contributes to the literature by quantifying effect sizes across clinical and structural outcomes, identifying potential factors associated with treatment variability, and integrating the findings within contemporary clinical and translational evidence. Below, the findings are discussed in four sections: overall therapeutic effects, factors influencing treatment outcomes, safety and translational implications, and limitations with future research directions.

Future research should prioritize the development of standardized treatment protocols, including optimized dosing strategies, uniform cell preparation and delivery methods, and harmonized outcome measurement frameworks. Such standardization is essential to reduce clinical and methodological heterogeneity, improve comparability across trials, and facilitate more robust evidence synthesis. In particular, the adoption of core outcome sets and consensus-based reporting guidelines would enhance reproducibility and strengthen the translational potential of MSC-based therapies for osteoarthritis.

### 4.1 Overall therapeutic benefits of stem cell therapy

#### 4.1.1 Overall therapeutic benefits of Stem cell therapy

Our results demonstrate that MSC treatment achieves consistent and clinically meaningful improvements across pain, function, and cartilage structure in OA. Compared with controls, MSC therapy significantly reduced pain (SMD = −1.31), lowered WOMAC scores (SMD = −0.78), increased cartilage volume (SMD = 0.91), and improved KOOS subscales including symptoms, activities of daily living, and sports/recreation. These findings are consistent with recent clinical trials and preclinical studies (Vega et al., 2015; Borakati et al., 2018; Iijima et al., 2018) and extend prior meta-analyses by including RCTs published up to 1 August 2025, and performing rigorous subgroup analyses to reduce heterogeneity.

Pathologically, OA is characterized by increased chondrocyte apoptosis, accelerated extracellular matrix degradation, and persistent low-grade inflammation (Chen et al., 2024; Tu et al., 2020). Conventional treatments—including NSAIDs, intra-articular hyaluronic acid injections, and arthroscopic debridement—mainly relieve symptoms but cannot restore cartilage structure or function (Correa and Lietman, 2017). In contrast, MSCs exert dual therapeutic effects: direct chondrogenic differentiation to form new cartilage-like tissue, and paracrine secretion of bioactive factors (e.g., TGF-β, IGF-1, IL-10) that modulate inflammation and improve the joint microenvironment (Zhao et al., 2020; He et al., 2023). This combination of symptomatic relief and structural repair supports a paradigm shift from palliative management to disease-modifying regenerative therapy for OA. Although the pooled analyses consistently favored MSC therapy across most outcomes, these findings should be interpreted with appropriate caution. Substantial residual heterogeneity remained in several analyses, and considerable variation existed in comparator interventions, patient characteristics, intervention protocols, study design, and follow-up duration across the included randomized controlled trials. Consequently, the reported pooled estimates represent the average comparative effectiveness of MSC therapy across diverse clinical settings and follow-up periods rather than superiority over any single comparator intervention or efficacy at a specific time point. Furthermore, because treatment effects were synthesized using standardized mean differences (SMDs) to accommodate different outcome instruments, the pooled estimates cannot be directly compared with established minimal clinically important difference (MCID) thresholds for measures such as WOMAC or KOOS. Therefore, although statistically significant improvements were observed, statistical significance should not be interpreted as definitive evidence that all observed benefits reached clinically meaningful levels.

### 4.2 Influencing factors and subgroup differences

Subgroup analyses suggested that several patient- and intervention-related factors may be associated with variability in the observed therapeutic effects of MSCs therapy in osteoarthritis. These factors can be broadly grouped into disease-related characteristics and treatment-related characteristics.

#### 4.2.1. Disease-related factors

MSC therapy appeared to be more effective in idiopathic osteoarthritis compared with post-traumatic disease. This may reflect differences in disease pathophysiology, as idiopathic OA is primarily driven by degenerative and inflammatory processes that may be more responsive to biological modulation, whereas post-traumatic OA is strongly influenced by mechanical instability and structural damage that may limit regenerative capacity (Cao et al., 2021; Colombini et al., 2019; Im and Kim, 2020; Harrell et al., 2019).

#### 4.2.2 Cell-related factors

Autologous MSCs demonstrated larger effect estimates than allogeneic MSCs, potentially due to improved immunological compatibility and reduced biological variability (Sackett et al., 2016; Al-Daccak and Charron, 2015). In addition, bone marrow-derived MSCs showed more favorable outcomes compared with adipose-derived MSCs, consistent with their higher chondrogenic potential and more favorable cytokine secretion profile (Mehlhorn et al., 2006; Jafri et al., 2020; Li et al., 2018; Perdisa et al., 2015).

#### 4.2.3 Delivery-related factors

Intra-articular injection without scaffold appeared to be associated with larger pooled effect estimates compared with scaffold-assisted implantation in some outcomes. However, this observation should be interpreted cautiously given substantial variability in scaffold materials, surgical techniques, and rehabilitation protocols across studies.

#### 4.2.4 Residual heterogeneity

Although these subgroup analyses suggest potential sources of variability in treatment effects, substantial heterogeneity remained unexplained across several outcomes. Additional factors, including baseline disease severity, MSC manufacturing and processing methods, follow-up duration, outcome measurement instruments, and comparator interventions, may also have contributed to the observed variability. However, these variables were inconsistently reported across the included studies, precluding formal evaluation through meta-regression or additional stratified analyses.

The particularly large effect observed in trauma-induced osteoarthritis should be interpreted cautiously due to the limited number of contributing studies, small sample sizes, and potential small-study effects, which may have influenced the stability of the pooled estimate.

#### 4.2.5 Global interpretation

Overall, these findings suggest that patient and treatment characteristics may influence observed treatment effects of MSC therapy; however, given the exploratory nature of subgroup analyses and the absence of formal interaction testing, these results should be interpreted as hypothesis-generating rather than definitive evidence of effect modification.

Current evidence is insufficient to define an optimal dosing strategy, as most studies were not designed for direct dose comparison. Therefore, reported dose ranges should be considered preliminary.

Similarly, precision-medicine stratification remains a promising but unvalidated conceptual framework and should be interpreted as exploratory rather than clinically established.

Collectively, these factors may contribute to variability in treatment effects but should be considered potential rather than definitive determinants of efficacy.

### 4.3 Safety profile and clinical potential

The present meta-analysis found no statistically significant difference in adverse event rates between MSCs therapy and control interventions (OR = 1.58, 95% CI: 0.72–3.47), suggesting a comparable short-term safety profile. Reported adverse events were generally mild and transient, most commonly including temporary joint swelling, discomfort, or short-term pain exacerbation, which typically resolved without additional intervention (Domper Arnal et al., 2022).

Consistent with previous studies, adverse events associated with intra-articular MSCs administration appear to be predominantly mild and self-limiting, with no clear evidence of increased risk of severe complications compared with control treatments (Riggle et al., 2024; Emadedin et al., 2015). However, available long-term safety data remain limited.

Long-term observational evidence, including the 10-year follow-up reported by Wong et al. (2013), did not identify signals of tumorigenesis, severe immune reactions, or accelerated joint degeneration. While these findings are reassuring, they are based on a limited number of long-term studies and should therefore be interpreted with caution.

Compared with conventional treatments such as long-term NSAID use and surgical interventions, MSCs therapy may offer a less invasive alternative with a potentially favorable safety profile; however, current evidence is insufficient to support definitive conclusions regarding its long-term comparative advantages.

Recent advances in MSCs research provide additional mechanistic insight into regenerative and immunomodulatory processes and complement the findings of this meta-analysis. Nevertheless, emerging approaches such as exosome-based therapies and precision regenerative medicine remain at an early investigational stage and are not yet supported by sufficient clinical evidence for routine clinical application in osteoarthritis (Barry and Murphy, 2004).

### 4.4 Limitations and future directions

#### 4.4.1 Exclusion of the 10-year Follow-Up Study by

Wong et al. (2013) reported extended 10-year follow-up outcomes from their randomized controlled trial cohort. However, the original 2-year trial data from the same study were already included in this meta-analysis. To avoid duplication of patient data and violation of independence assumptions in meta-analysis, the extended follow-up results were not included.

#### 4.4.2 Updated limitations based on recent advances

Despite the inclusion of recent randomized controlled trials, several important limitations should be considered when interpreting the findings of this meta-analysis.

First, substantial clinical and methodological heterogeneity remains a major limitation. Variations in baseline osteoarthritis severity, stem cell source (bone marrow, adipose tissue, or umbilical cord), manufacturing and processing protocols, dosage, delivery methods, comparator interventions, rehabilitation strategies, outcome measurement instruments, and follow-up duration were observed across the included studies. Although random-effects models and predefined subgroup analyses were applied, considerable residual heterogeneity remained unexplained in several outcomes, which may reduce the precision and generalizability of pooled estimates.

Second, limited long-term evidence is available. Most included randomized controlled trials reported follow-up durations of 6–24 months, with only a small number extending beyond 5 years. Therefore, it remains uncertain whether short-to mid-term improvements in pain, function, and cartilage structure translate into sustained long-term benefits or reduced rates of joint replacement.

Third, mechanistic understanding of MSC therapy remains incomplete. Although paracrine signaling and immunomodulatory effects are considered the primary mechanisms, the relative contributions of direct chondrogenic differentiation, extracellular vesicle signaling, and metabolic regulation remain unclear and require further translational investigation.

Fourth, concerns remain regarding small-study effects and potential publication bias. Although funnel plots were used for assessment, their interpretability is limited when the number of included studies is small and heterogeneity is substantial. Therefore, publication bias assessments should be interpreted with caution.

Fifth, the use of standardized mean differences (SMDs) limited direct clinical interpretability, as effect estimates could not be compared with established minimal clinically important difference (MCID) thresholds for individual outcome instruments such as WOMAC and KOOS.

Finally, additional analyses such as meta-regression were not performed due to the limited number of included studies and insufficient reporting of key covariates, which restricted robust exploration of heterogeneity sources.

Collectively, these factors highlight important limitations in the certainty, interpretability, and generalizability of the current evidence base, and underscore the need for future large-scale, well-designed randomized controlled trials with standardized protocols and long-term follow-up.

#### 4.4.3 Novel contributions relative to Existing Systematic reviews

Numerous systematic reviews and meta-analyses have previously evaluated MSCs therapy for articular cartilage lesions, including a recent overview by (Sousa et al., 2025), which confirmed the potential of MSC therapy while also highlighting substantial heterogeneity and low-to-very low certainty of evidence across existing studies. In this context, the present study provides several methodological and analytical contributions.

First, this meta-analysis included only randomized controlled trials, thereby excluding observational studies, case series, and preclinical evidence. This restriction reduces risk of bias and strengthens the internal validity of the pooled estimates.

Second, predefined subgroup analyses were conducted to explore potential sources of heterogeneity, including disease etiology, stem cell source, tissue origin, and delivery method. These analyses provide exploratory evidence of factors that may be associated with variability in treatment effects; however, they were not designed to establish causal or definitive effect modification.

Third, this study integrates recent clinical and translational evidence, including data on long-term outcomes, manufacturing considerations, and emerging therapeutic approaches such as exosome-based and precision regenerative strategies, to provide a more updated synthesis of the current evidence landscape.

Finally, despite the use of predefined subgroup analyses, substantial residual heterogeneity remained across multiple outcomes. These findings underscore the complexity and variability of the available evidence base. Therefore, subgroup results should be interpreted as hypothesis-generating rather than confirmatory, and require validation in future individual patient data meta-analyses and well-designed head-to-head randomized controlled trials.

#### 4.4.4 Priorities for Future clinical research

1. Standardize cell products. Enforce ISCT criteria for MSC characterization, control passage numbers (P2–P4), and ensure consistent viable cell dosing to reduce between-study heterogeneity.
2. Extend follow-up duration. Future randomized controlled trials should include long-term clinical and imaging follow-up (≥3–5 years) to evaluate durability of effects and potential disease-modifying outcomes.
3. Develop precision-medicine strategies. Patient stratification based on osteoarthritis etiology, disease severity, and inflammatory phenotype may help optimize treatment selection; however, these approaches remain investigational and require prospective validation.
4. Explore combination therapies. MSC-based therapies combined with PRP, biomechanical stabilization, or anti-inflammatory agents warrant further investigation, particularly in post-traumatic and advanced osteoarthritis.
5. Establish international registries. Real-world evidence from standardized registries is needed to evaluate long-term safety, effectiveness, and cost-effectiveness.

## 5 Conclusion

This systematic review and meta-analysis suggests that MSCs therapy is associated with improvements in pain, functional outcomes, and cartilage-related measures in osteoarthritis, without a significant increase in adverse events compared with control interventions.

However, the certainty of the evidence is limited by substantial between-study heterogeneity, variability in intervention protocols, and insufficient long-term follow-up data. Therefore, these findings should be interpreted as supportive but not definitive evidence of MSC efficacy.

Subgroup analyses suggest potential variability in treatment response according to disease etiology and stem cell source; however, these findings are exploratory and should not be interpreted as definitive evidence of superiority among treatment strategies.

Further large-scale, well-designed randomized controlled trials with standardized protocols and long-term follow-up are required to clarify the efficacy, safety, and potential disease-modifying effects of MSC therapy.
