# Supplementary material for: Exploring the effectiveness and safety of stem cell therapy for repair of cartilage defects: a meta-analysis of randomized controlled trials
Source: Front Cell Dev Biol. 2026 Aug 7;14:1880031. doi: 10.3389/fcell.2026.1880031 (PMC13493546; doi:10.3389/fcell.2026.1880031)
Supplement: Supplementary file 1 [file Image1.pdf]

Supplement Figure 1. Funnel plot of pain outcomes

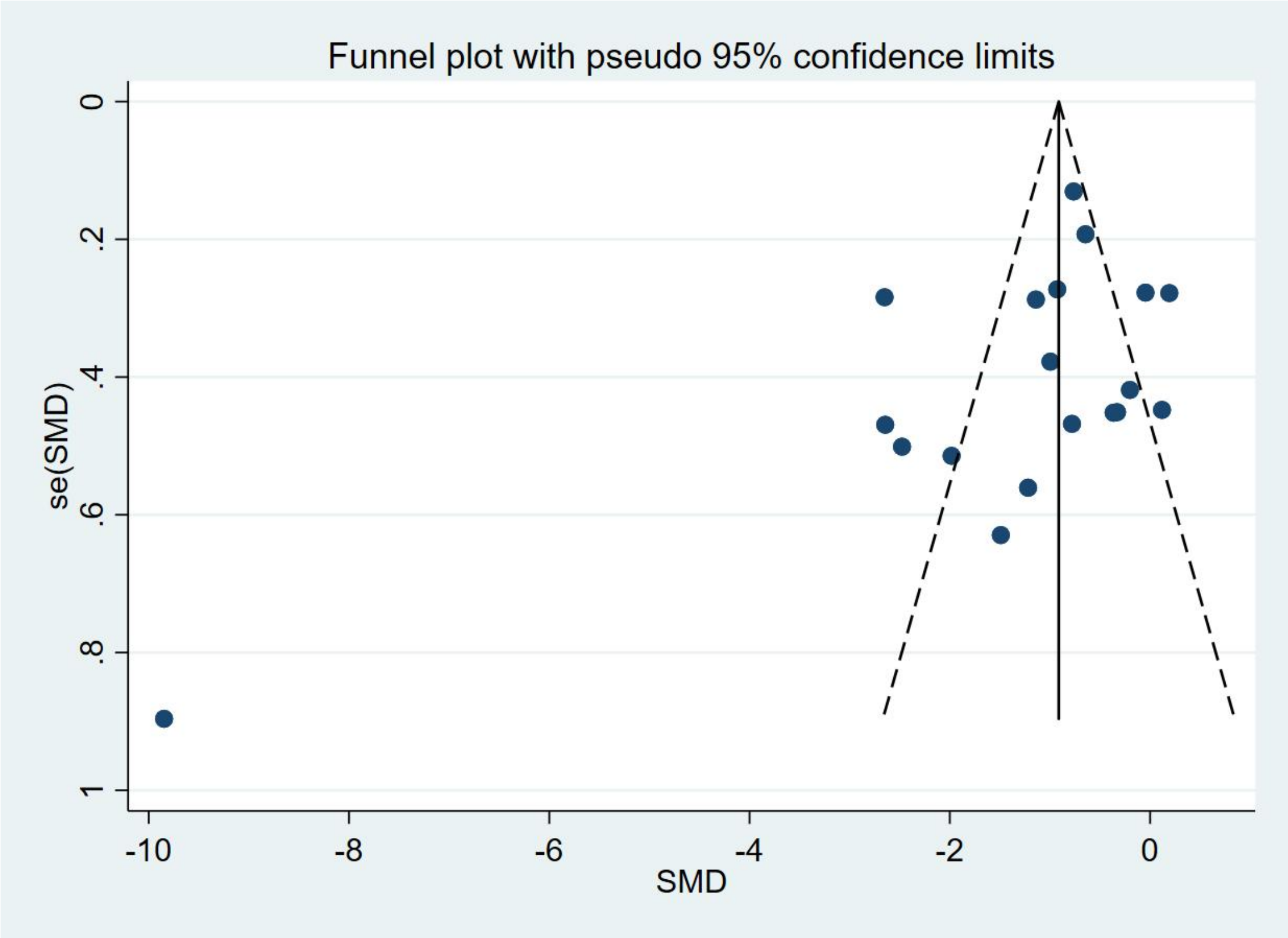

Note: This funnel plot illustrates potential publication bias for pain outcomes across the included randomized controlled trials.

Each point represents an individual study, plotted as the standardized mean difference (SMD) against its standard error. The solid vertical line indicates the pooled effect estimate, while the diagonal dashed lines represent the pseudo 95% confidence limits.

Visual inspection of funnel plot symmetry was used to assess potential publication bias and small-study effects. Although the distribution of studies appears relatively symmetrical, interpretation should be made with caution due to the limited number of included studies and the presence of substantial between-study heterogeneity, which may reduce the reliability of funnel plot asymmetry assessment.

Abbreviations: SMD, standardized mean difference.

Supplement Figure 2. Funnel plot of WOMAC outcomes

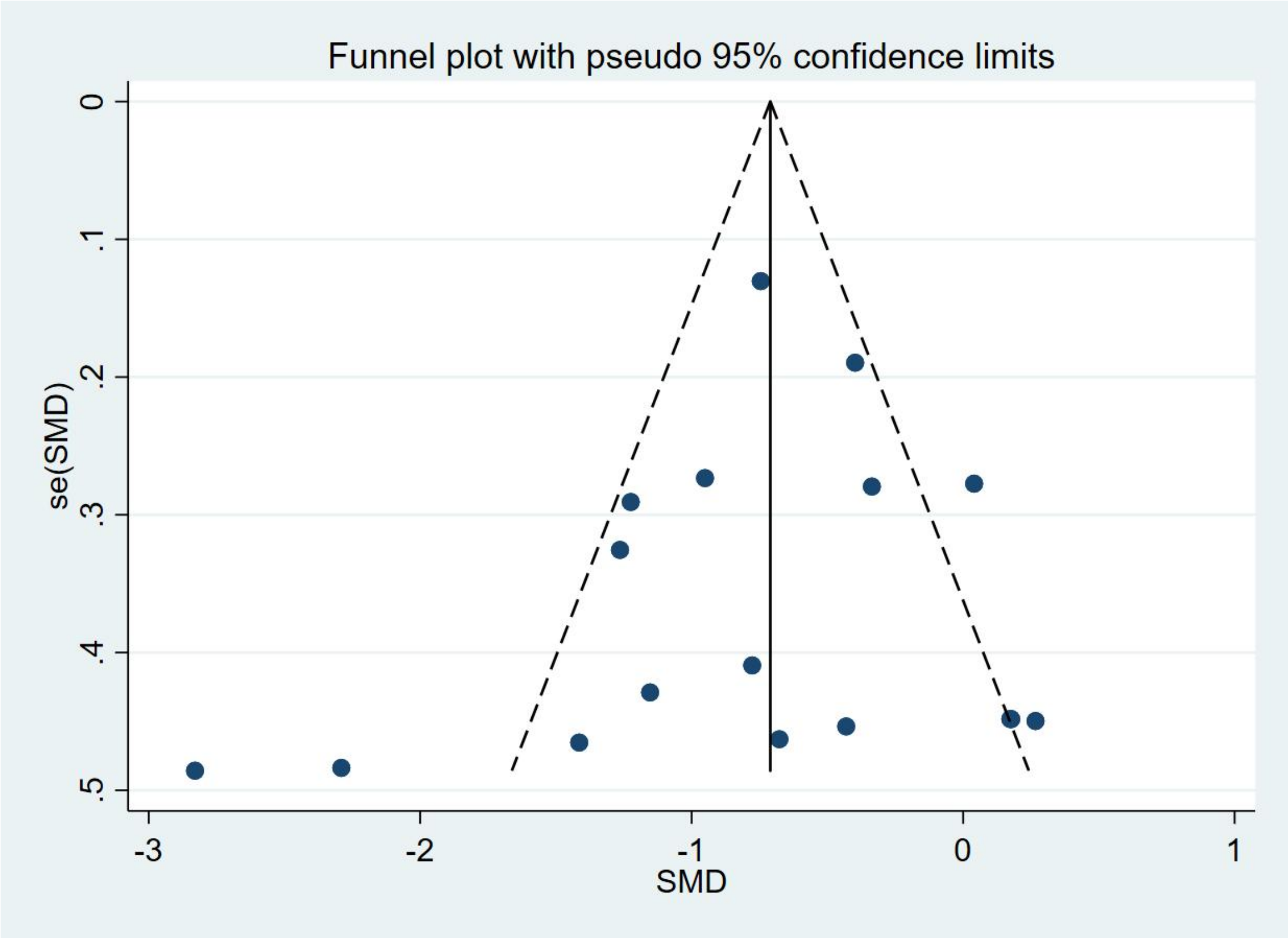

Note: This funnel plot displays the assessment of potential publication bias for WOMAC outcomes across the included randomized controlled trials.

Each point represents an individual study, plotted as the standardized mean difference (SMD) against its standard error. The solid vertical line indicates the pooled effect estimate, and the diagonal dashed lines represent the pseudo 95% confidence limits.

Visual inspection of funnel plot symmetry was used to evaluate potential publication bias and small-study effects. The distribution of studies appears generally symmetrical around the pooled estimate; however, interpretation should be made with caution due to the relatively small number of included studies and substantial between-study heterogeneity, which may limit the reliability of funnel plot asymmetry assessment.

Abbreviations: SMD, standardized mean difference; WOMAC, Western Ontario and McMaster Universities Osteoarthritis Index.

Supplement Figure 3. Funnel plot of cartilage volume outcomes

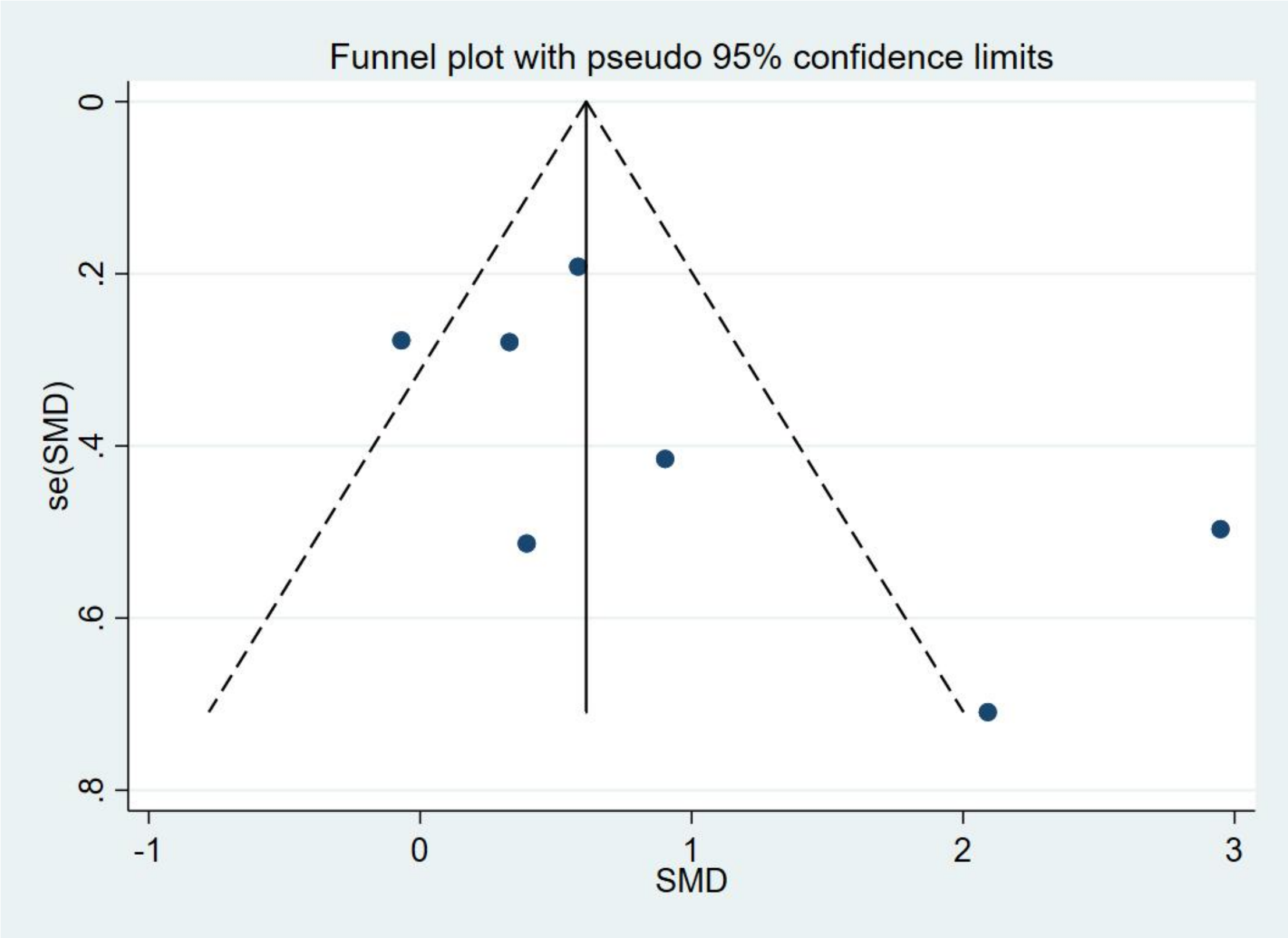

Note: This funnel plot illustrates the assessment of potential publication bias for cartilage volume outcomes across the included randomized controlled trials.

Each point represents an individual study, plotted as the standardized mean difference (SMD) against its standard error. The solid vertical line indicates the pooled effect estimate, and the diagonal dashed lines represent the pseudo 95% confidence limits.

Visual inspection of funnel plot symmetry was used to evaluate potential publication bias and small-study effects. The distribution of studies appears moderately asymmetric, particularly with limited representation on one side of the funnel, suggesting the possibility of small-study effects; however, interpretation should be made with caution due to the small number of included studies and substantial between-study heterogeneity, which may limit the reliability of asymmetry assessment.

Abbreviations: SMD, standardized mean difference.

Supplement Figure 4. Funnel plot of complications

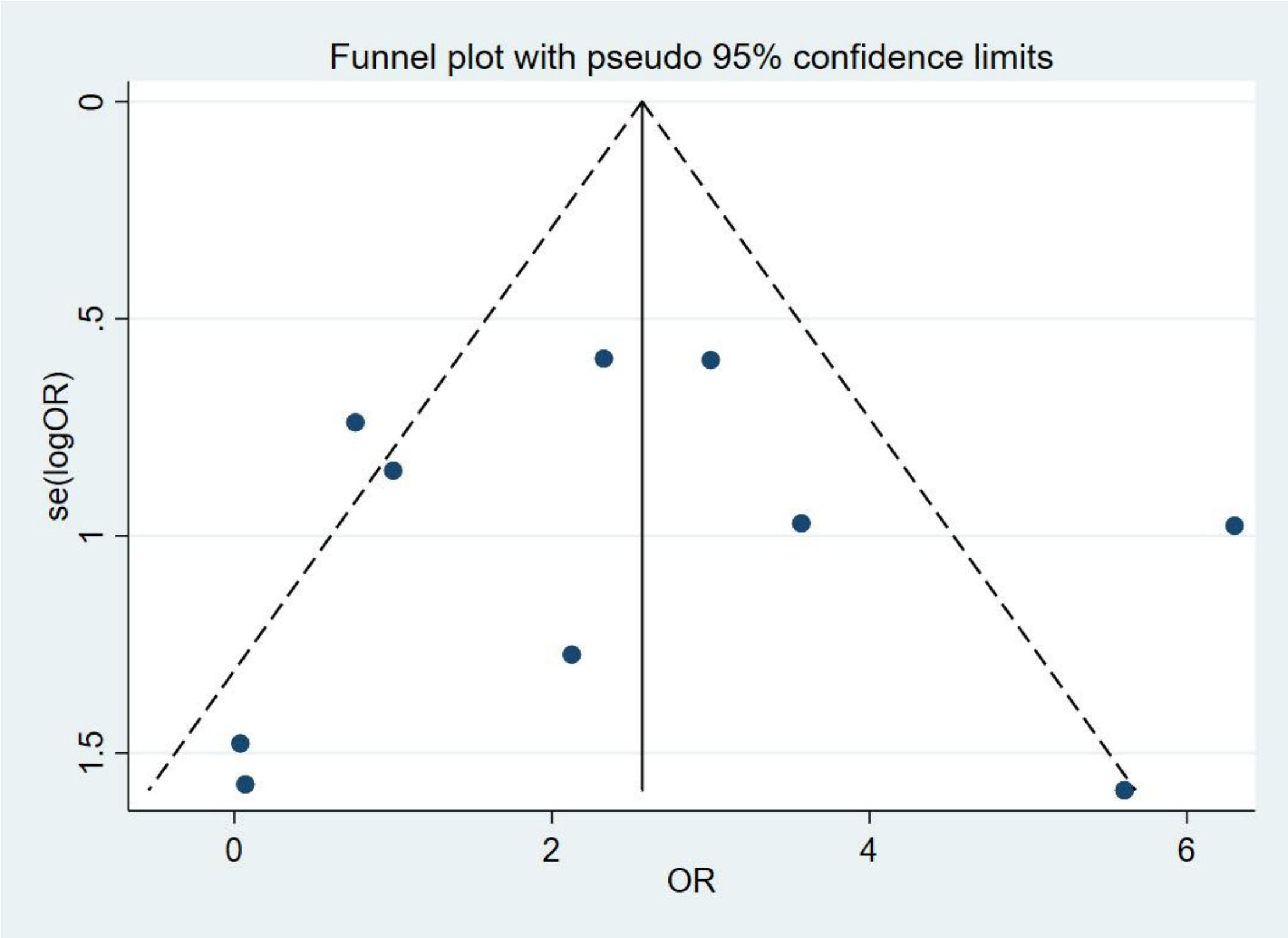

Note: This funnel plot illustrates the assessment of potential publication bias for complication outcomes across the included randomized controlled trials.

Each point represents an individual study, plotted as the log odds ratio (logOR) against its standard error. The solid vertical line indicates the pooled effect estimate, and the diagonal dashed lines represent the pseudo 95% confidence limits.

Visual inspection of funnel plot symmetry was used to evaluate potential publication bias and small-study effects. The distribution of studies appears moderately asymmetric, with some dispersion at the lower precision range; however, interpretation should be made with caution due to the limited number of included studies and between-study heterogeneity, which may affect the reliability of funnel plot asymmetry assessment.

Abbreviations: OR, odds ratio; logOR, logarithm of odds ratio.

Supplementary Figure 5. Forest plot of KOOS-S (Symptoms) outcomes

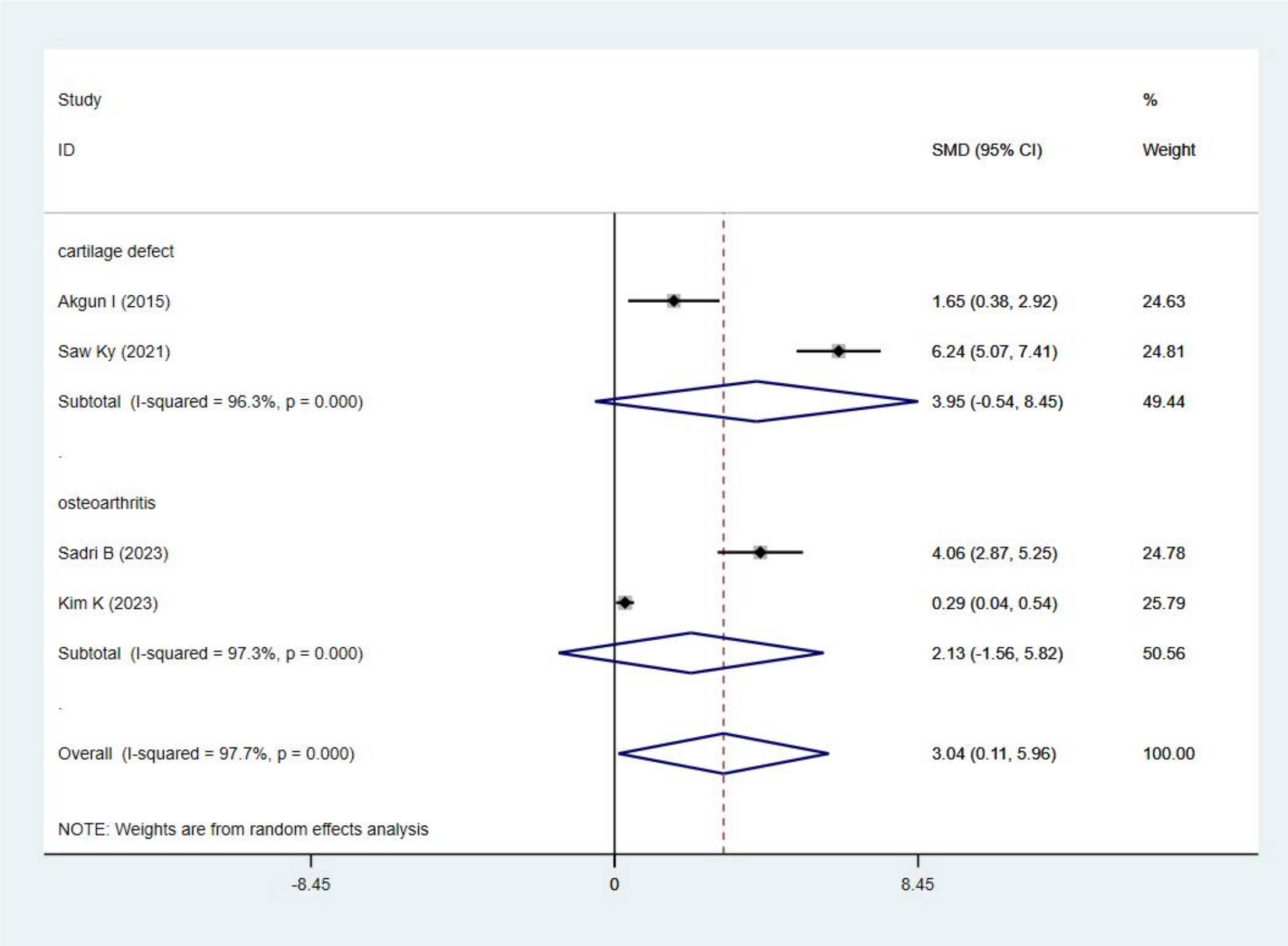

Note: This forest plot presents the pooled effect estimates for KOOS-S (Symptoms) outcomes comparing mesenchymal stem cell (MSC) therapy with control interventions.

Individual study effect sizes are expressed as standardized mean differences (SMD) with 95% confidence intervals (CI). The diamond represents the pooled effect estimate, with its width indicating the overall confidence interval. Subgroup analyses were performed according to disease classification (cartilage defect vs osteoarthritis), and subgroup-specific pooled estimates are shown. The vertical solid line represents the line of no effect.

A random-effects model was used due to substantial between-study heterogeneity. The I^2 statistic and p values are reported to quantify heterogeneity across studies and subgroups.

Abbreviations: SMD, standardized mean difference; CI, confidence interval; KOOS-S, Knee injury and Osteoarthritis Outcome Score–Symptoms.

Supplement Figure 6. Funnel plot of KOOS-S (Symptoms) outcomes

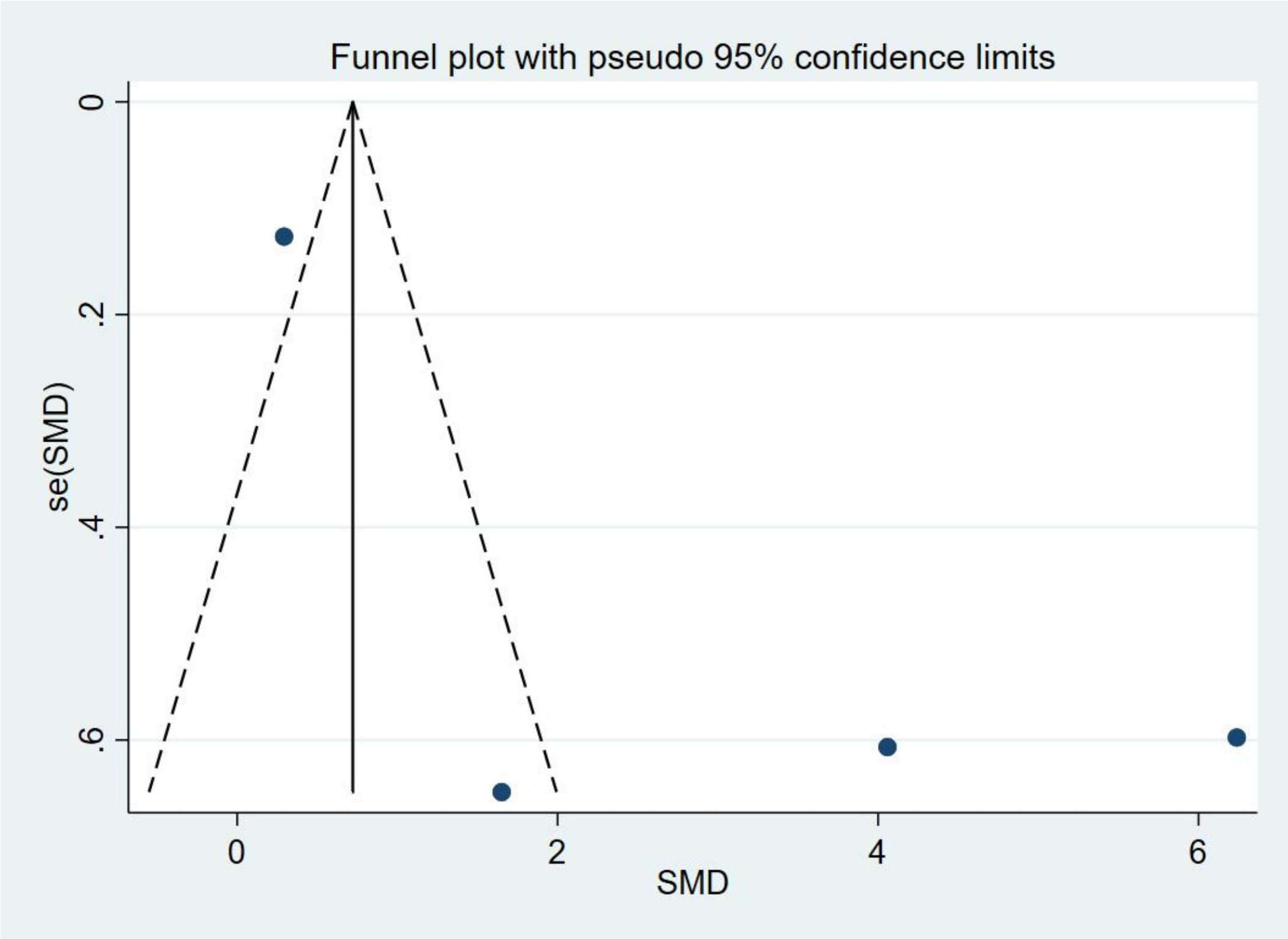

Note: This funnel plot assesses potential publication bias in studies reporting KOOS-S (Symptoms) outcomes comparing mesenchymal stem cell (MSC) therapy with control interventions.

Each dot represents an individual study, plotted by effect size (standardized mean difference, SMD) on the horizontal axis and standard error on the vertical axis. The solid vertical line indicates the pooled effect estimate, while the dashed lines represent pseudo 95% confidence limits around the summary effect. In the absence of publication bias, studies are expected to be symmetrically distributed around the pooled estimate within the triangular funnel region.

Visual inspection of the plot suggests potential asymmetry; however, interpretation should be cautious due to the small number of included studies and substantial between-study heterogeneity.

Supplement Figure 7. Forest plot of KOOS-ADL (Activities of Daily Living) outcomes

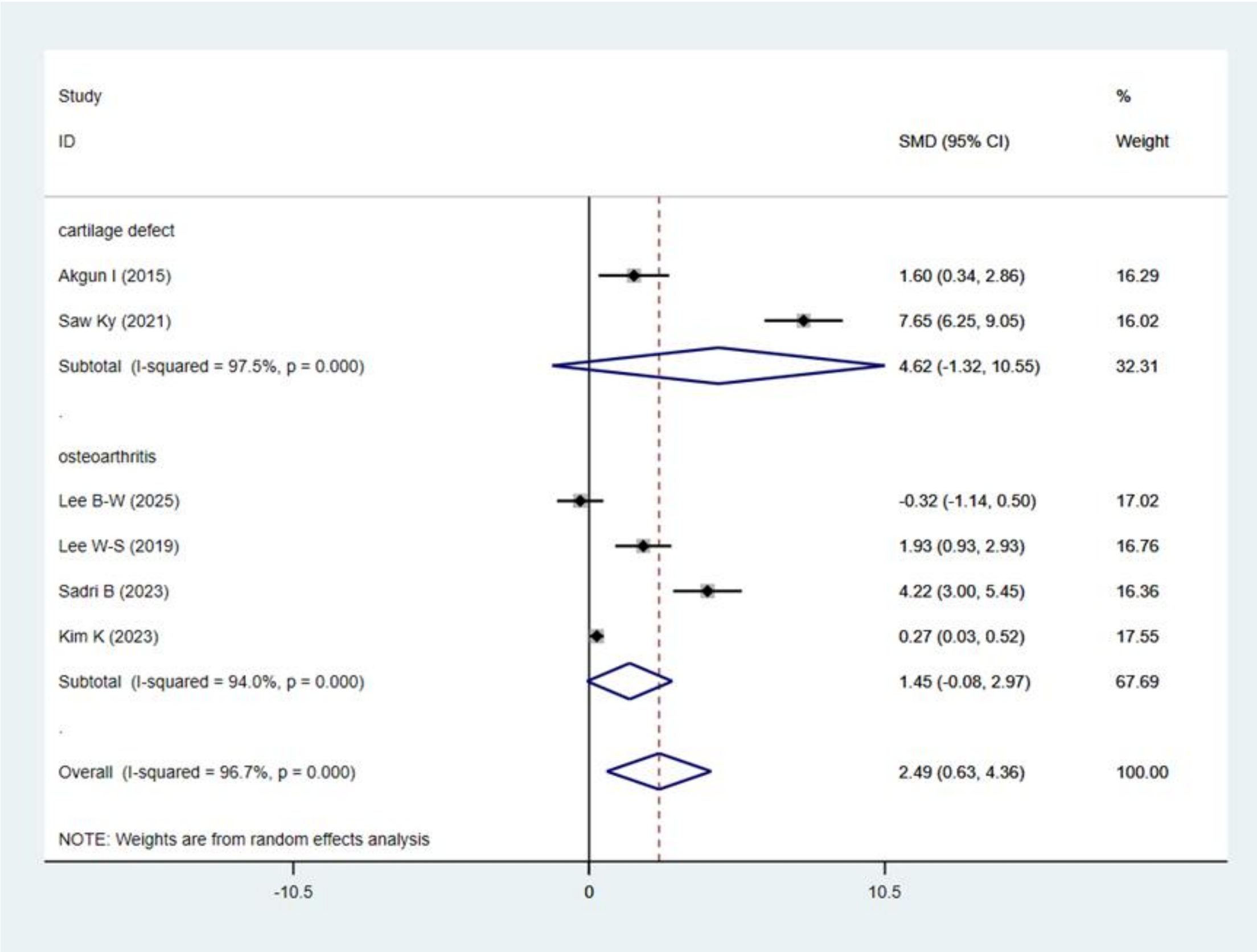

Note: This forest plot summarizes the effect of mesenchymal stem cell (MSC) therapy compared with control interventions on KOOS-ADL (Activities of Daily Living) scores across included randomized controlled trials.

Individual study effect estimates (black squares) and corresponding 95% confidence intervals (horizontal lines) are presented. The size of each square reflects the relative weight of each study in the meta-analysis. The pooled effect estimate is represented by the blue diamond, with the center indicating the overall standardized mean difference (SMD) and the width representing the 95% confidence interval. The vertical solid line denotes the line of no effect (SMD = 0).

Subgroup analyses were conducted according to disease type (cartilage defect vs. osteoarthritis), with corresponding pooled estimates shown for each subgroup. Substantial heterogeneity was observed across studies ($I^2 > 90\%$), and therefore results should be interpreted with caution.

Supplement Figure 8. Funnel plot of KOOS-ADL (Activities of Daily Living) outcomes

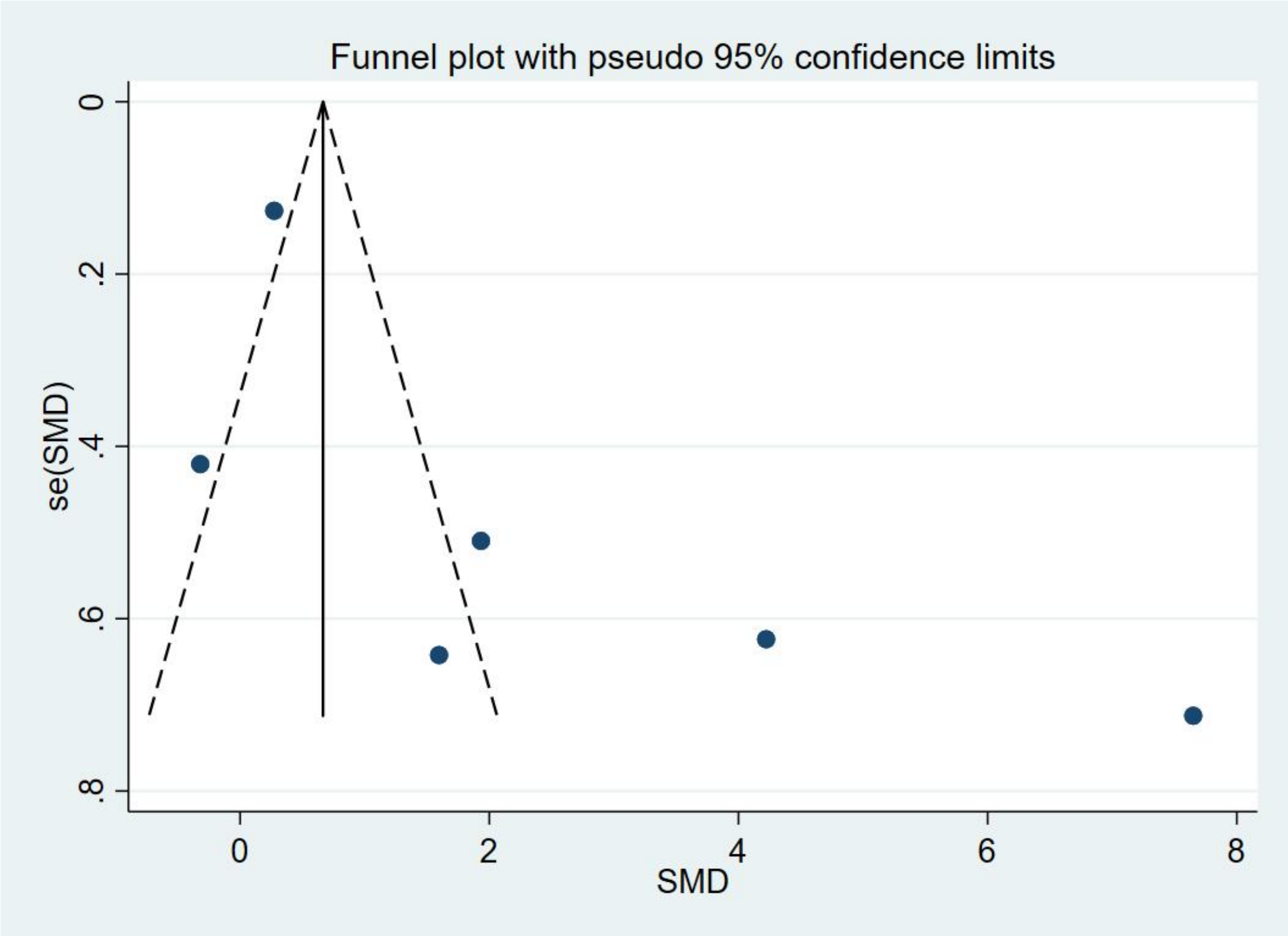

Note: This funnel plot evaluates potential publication bias for studies reporting KOOS-ADL (Activities of Daily Living) outcomes in patients receiving mesenchymal stem cell (MSC) therapy compared with control interventions.

Each point represents an individual study, plotted according to the effect size (standardized mean difference, SMD) on the horizontal axis and its corresponding standard error on the vertical axis. The solid vertical line indicates the pooled effect estimate, while the dashed lines represent pseudo 95% confidence limits around the summary effect. The triangular region illustrates the expected distribution of studies in the absence of publication bias.

Visual inspection suggests possible asymmetry in the distribution of studies; however, interpretation should be made with caution due to the small number of included studies and substantial between-study heterogeneity.

Supplement Figure 9. Forest plot of KOOS-SR (Sports and Recreation) outcomes

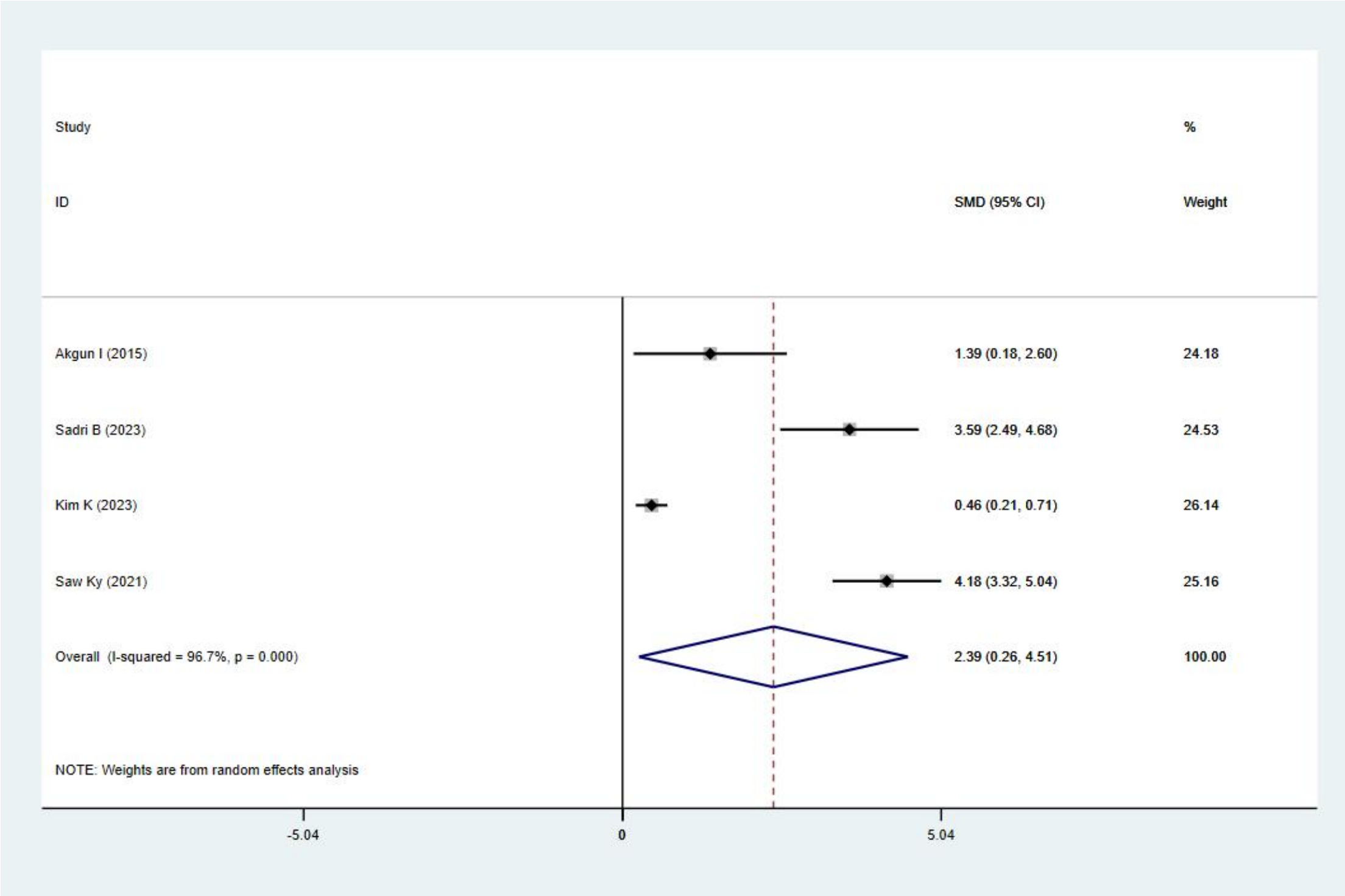

Note: This forest plot presents the effect of mesenchymal stem cell (MSC) therapy compared with control interventions on KOOS-SR (Sports and Recreation) outcomes across included randomized controlled trials.

Individual study estimates (black squares) with corresponding 95% confidence intervals (horizontal lines) are shown. The size of each square reflects the weight of each study in the meta-analysis. The pooled effect estimate is represented by the blue diamond, with the center indicating the overall standardized mean difference (SMD) and the width representing the 95% confidence interval. The vertical solid line indicates the line of no effect (SMD = 0).

Substantial heterogeneity was observed among studies ($I^2 > 95\%$), suggesting considerable variability in treatment effects across trials. Therefore, results should be interpreted with caution, and subgroup findings should be considered exploratory.

Supplement Figure 10. Funnel plot of KOOS-SR (Sports and Recreation) outcomes

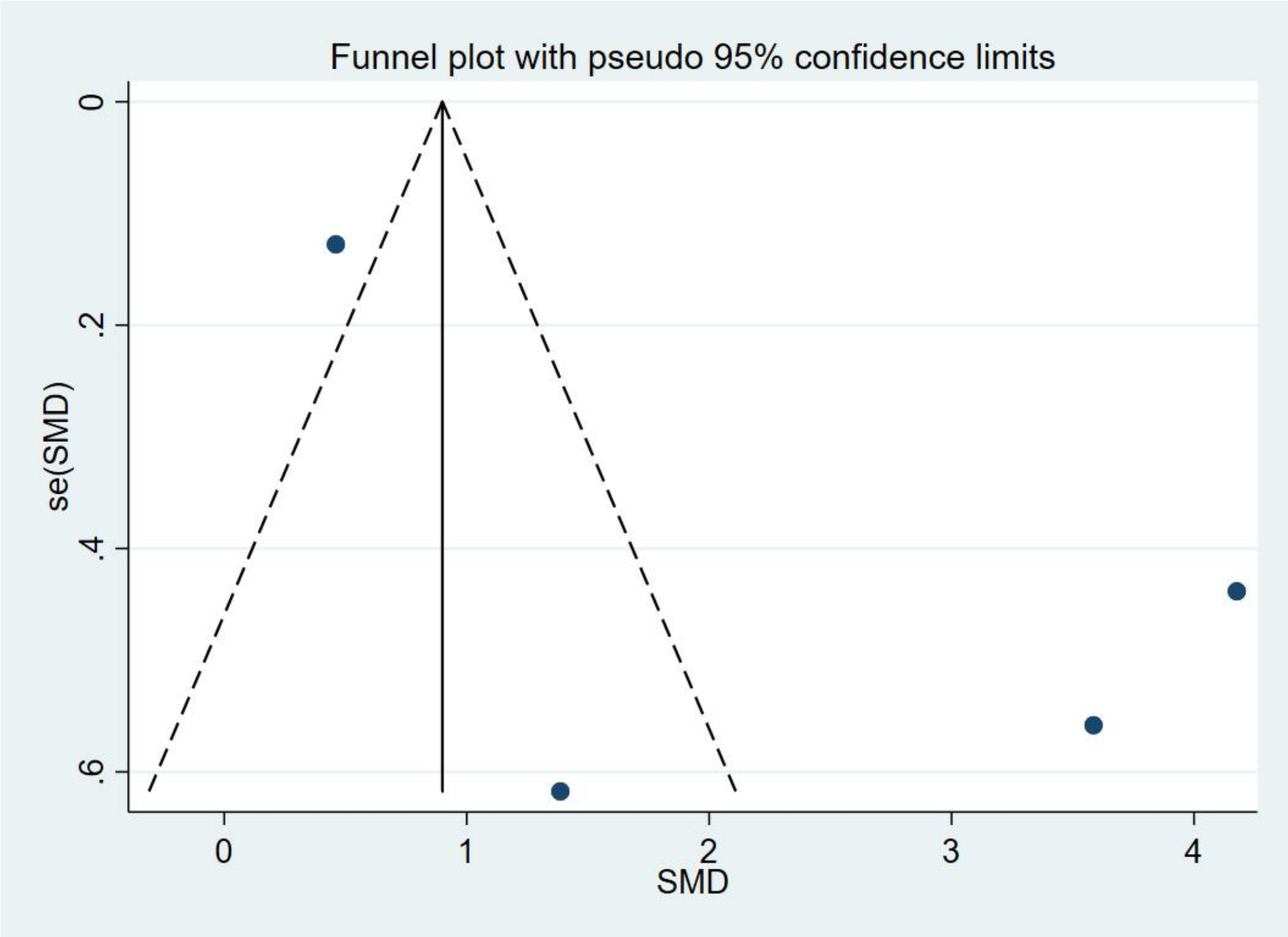

Note: This funnel plot evaluates potential publication bias in studies reporting KOOS-SR (Sports and Recreation) outcomes in patients receiving mesenchymal stem cell (MSC) therapy compared with control interventions.

Each dot represents an individual study, plotted according to the effect size (standardized mean difference, SMD) on the horizontal axis and its corresponding standard error on the vertical axis. The solid vertical line indicates the pooled effect estimate, while the dashed lines represent pseudo 95% confidence limits around the summary effect. The triangular region represents the expected distribution of studies in the absence of publication bias.

Visual inspection suggests possible asymmetry in the distribution of studies; however, interpretation should be made with caution due to the very small number of included studies and substantial between-study heterogeneity.
