# Supplementary material for: Exploring the effectiveness and safety of stem cell therapy for repair of cartilage defects: a meta-analysis of randomized controlled trials
Source: Front Cell Dev Biol. 2026 Aug 7;14:1880031. doi: 10.3389/fcell.2026.1880031 (PMC13493546; doi:10.3389/fcell.2026.1880031)
Supplement: Supplementary file 2 [file Table1.pdf]

Supplement Table 1. Risk of bias assessment of the included randomized controlled trials using the Cochrane Risk of Bias 2.0 tool

Study ID	Random sequence generation	Allocationconcealment	Blinding of participants	Blinding of operator	Blinding of outcome assessment	Incomplete outcome data (report)	Selective reporting(report)	Other bias
Akgun I 2015	Y	Y	N	N	Y	N	N	N
Bolandnazar NS 2024	Y	Y	Y	Y	Y	N	N	N
Garza J.R. 2020	PY	Y	Y	Y	Y	N	N	N
Gupta P.K 2016	PY	Y	Y	Y	Y	N	N	N
Hernigou P 2018	Y	Y	N	N	N	N	PY	N
Kim J-H 2022	PY	Y	N	N	Y	N	N	N
Lee B-W 2025	PY	Y	Y	Y	Y	N	N	N
Lee W-S 2019	PY	Y	Y	Y	Y	N	N	N
Lu L-J 2019	Y	Y	Y	Y	Y	N	N	N
Sadri B 2023	Y	Y	Y	Y	Y	N	N	N
Sun H 2024	Y	Y	N	N	Y	N	N	N
Wang Y 2017	Y	Y	Y	N	Y	N	N	N
Zhang S 2022	Y	Y	N	N	Y	N	N	N
Zhang Y 2022	Y	Y	Y	N	Y	N	N	N
Zhou Y 2021	Y	Y	N	N	Y	N	N	N
Masoumeh Z 2025	Y	Y	Y	PN	Y	N	N	N
Hong Z 2018	Y	Y	Y	N	Y	N	N	N
Kim K 2023	Y	Y	Y	N	Y	N	N	N
Qiao Z 2020	Y	Y	Y	N	Y	N	N	N
Thomas C 2014	PY	Y	Y	Y	Y	N	N	N
Vega A 2015	PY	Y	Y	Y	Y	N	N	N
Wong KL 2013	Y	Y	N	N	Y	N	N	N
Saw Ky 2013	Y	Y	N	N	Y	N	N	N
Saw Ky 2021	Y	Y	N	N	PN	N	PY	N

Note: This table summarizes the risk of bias assessment for each included randomized controlled trial across the following domains: random sequence generation, allocation concealment, blinding of participants, blinding of personnel (operators), blinding of outcome assessment, incomplete outcome data, selective reporting, and other sources of bias.

Risk of bias judgments were classified as “Yes (Y),” “Probably Yes (PY),” “Probably No (PN),” or “No (N)” according to the Cochrane Risk of Bias 2.0 framework. “Y” indicates low risk of bias, “PY” indicates probably low risk, “PN” indicates probably high risk, and “N” indicates high risk of bias.

Abbreviations: Y, yes (low risk of bias); PY, probably yes (probably low risk); PN, probably no (probably high risk); N, no (high risk of bias).

Supplement Table 2. Subgroup analyses of pain outcomes

	Articlecount	n	SMD(95%CI)	Weight	I^2	P
**Source**						
Autologous	10	543	-1.88（-2.82，-0.95）	52.36	94.9%	0.000
Allogeneic	9	465	-0.80（-1.23，-0.38）	47.64	68.9%	0.001
**Location**						
Bone marrow	8	245	-1.00（-1.84，-0.16）	41.47	87.3%	0.000
Adipose	8	628	-1.02（-1.41，-0.62）	44.75	77.1%	0.000
**Stent**						
Yes	1	14	-1.49（-2.72，-0.26）	4.5	NA	NA
No	18	994	-1.30（1.83，-0.78）	95.50	91.7%	0.000

Note: This table presents subgroup analyses of pain outcomes based on stem cell source (autologous vs allogeneic), tissue origin (bone marrow vs adipose-derived), and delivery approach (with vs without scaffold implantation).

Effect estimates are reported as standardized mean differences (SMD) with 95% confidence intervals (CI). The number of included studies, total sample size, study weights, heterogeneity (I^2), and p values for subgroup analyses are also presented.

Random-effects models were used for all analyses. Heterogeneity within subgroups was assessed using the I^2 statistic, and p values indicate between-study heterogeneity within each subgroup.

Abbreviations: SMD, standardized mean difference; CI, confidence interval; I^2, heterogeneity statistic; NA, not applicable.

Supplement Table 3. Subgroup analyses of WOMAC outcomes

	**Articlecount**	**n**	**SMD(95%CI)**	**Weight**	**I^2**	**P**
**Source**						
Autologous	10	451	-0.77（-1.17，-0.38）	60.14	73.4%	0.000
Allogeneic	7	425	-0.79（-1.36，-0.22）	39.86	80.0%	0.000
**Location**						
Bone marrow	5	110	-0.60（-1.46，0.26）	25	78.2%	0.001
Adipose	11	714	-0.92（-1.26，-0.59）	68.17	73.3%	0.000

Note: This table presents subgroup analyses of WOMAC outcomes based on stem cell source (autologous vs allogeneic) and tissue origin (bone marrow-derived vs adipose-derived).

Effect estimates are expressed as standardized mean differences (SMD) with 95% confidence intervals (CI). The number of included studies, total sample size, study weights, heterogeneity (I^2), and p values are reported for each subgroup.

All analyses were conducted using a random-effects model. Within-subgroup heterogeneity was assessed using the I^2 statistic, and reported p values reflect statistical heterogeneity within each subgroup.

Abbreviations: SMD, standardized mean difference; CI, confidence interval; I^2, heterogeneity statistic.

Supplement Table 4. Subgroup analyses of cartilage volume outcomes

	Articlecount	n	SMD(95%CI)	Weight	I^2	P
**Source**						
Autologous	5	259	0.55（0.09，1.01）	74.45	62.6%	0.030
Allogeneic	2	53	1.67（-0.83，4.18）	25.55	92.2%	0.000
**Location**						
Bone marrow	4	229	2.09（0.70，3.48）	61.17	86.8%	0.000
Adipose	1	17	1.00（0.24，1.96）	9.75	NA	NA
**Stent**						
Yes	1	14	-1.49（-2.72，-0.26）	9.75	NA	NA
No	6	298	0.78（0.14，1.41）	90.25	83.2%	0.000

Note: This table presents subgroup analyses of cartilage volume outcomes based on stem cell source (autologous vs allogeneic), tissue origin (bone marrow-derived vs adipose-derived), and delivery approach (with vs without scaffold implantation).

Effect estimates are expressed as standardized mean differences (SMD) with 95% confidence intervals (CI). The number of included studies, total sample size, study weights, heterogeneity (I^2), and p values are reported for each subgroup.

All analyses were conducted using random-effects models. Within-subgroup heterogeneity was assessed using the I^2 statistic, and reported p values reflect statistical heterogeneity within each subgroup.

Abbreviations: SMD, standardized mean difference; CI, confidence interval; I^2, heterogeneity statistic; NA, not applicable.

Supplement Table 5. Subgroup analyses of KOOS-S (Symptoms) outcomes

	Articlecount	n	SMD(95%CI)	Weight	I^2	P
**Source**						
Autologous	2	83	3.95（-0.54，8.45）	49.44	96.3%	0.000
Allogeneic	2	288	2.13（-1.56，5.82）	50.56	97.3%	0.000
**Location**						
Peripheral blood	1	14	6.24（5.07，7.41）	24.81	NA	NA
Adipose	2	288	2.13（-1.56，5.82）	50.56	97.3%	0.000
Bone synovial tissue	1	69	1.65（0.38，2.92）	24.63	NA	NA
**Stent**						
Yes	1	14	1.65（0.38，2.92）	24.63	NA	NA
No	3	357	3.50（-0.47，7.47）	75.37	98.4%	0.000

Note: This table presents subgroup analyses of KOOS-S (Symptoms) outcomes according to stem cell source (autologous vs allogeneic), tissue origin (peripheral blood, adipose-derived, and bone synovial tissue), and delivery approach (with vs without scaffold implantation).

Effect estimates are reported as standardized mean differences (SMD) with 95% confidence intervals (CI). The number of included studies, total sample size, study weights, heterogeneity (I^2), and p values are presented for each subgroup.

All analyses were conducted using random-effects models. Within-subgroup heterogeneity was assessed using the I^2 statistic, and reported p values reflect heterogeneity within each subgroup.

Abbreviations: SMD, standardized mean difference; CI, confidence interval; I^2, heterogeneity statistic; NA, not applicable.

Supplement Table 6. Subgroup analyses of KOOS-ADL (Activities of Daily Living) outcomes

	Articlecount	n	SMD(95%CI)	Weight	I^2	P
**Source**						
Autologous	3	107	3.70（0.17，7.24）	49.07	96.1%	0.000
Allogeneic	3	311	1.30（-0.60，3.20）	50.93	95.2%	0.000
**Location**						
Peripheral blood	1	14	7.65（6.25，9.05）	16.02	NA	NA
Adipose	3	312	2.08（-0.17，4.33）	50.67	95.7%	0.000
Bone synovial tissue	1	69	1.60（0.34，2.86）	16.29	NA	NA
Bone marrow	1	23	-0.32（-1.14，0.50）	17.02	NA	NA
**Stent**						
Yes	1	14	1.60（0.34，2.86）	16.29	NA	NA
No	5	404	2.68（0.50，4.85）	83.71	97.3%	0.000

Note: This table presents subgroup analyses of KOOS-ADL (Activities of Daily Living) outcomes according to stem cell source (autologous vs allogeneic), tissue origin (peripheral blood, adipose-derived, bone synovial tissue, and bone marrow-derived), and delivery approach (with vs without scaffold implantation).

Effect estimates are reported as standardized mean differences (SMD) with 95% confidence intervals (CI). The number of included studies, total sample size, study weights, heterogeneity (I^2), and p values are provided for each subgroup.

All analyses were performed using random-effects models. Within-subgroup heterogeneity was assessed using the I^2 statistic, and reported p values correspond to heterogeneity within each subgroup.

Abbreviations: SMD, standardized mean difference; CI, confidence interval; I^2, heterogeneity statistic; NA, not applicable.

Supplement Table 7. Subgroup analyses of KOOS-SR (Sports and Recreation) outcomes

	Articlecount	n	SMD(95%CI)	Weight	I^2	P
**Source**						
Autologous	2	83	2.82（0.08，5.55）	49.33	92.6%	0.000
Allogeneic	2	288	1.98（-1.09，5.04）	50.67	96.6%	0.000
**Location**						
Peripheral blood	1	69	4.18（3.32，5.04）	25.16	96.6%	0.000
Adipose	2	288	1.98（-1.09，5.04）	50.67	NA	NA
Bone synovial tissue	1	14	1.39（0.18，2.60）	24.18	NA	NA
**Stent**						
Yes	1	14	1.39（0.18，2.60）	24.18	NA	NA
No	3	357	2.71（-0.06，5.48）	75.82	97.8%	0.000
**Disease type**						
Endogenous factors	2	288	1.98（-1.09，5.04）	50.67	96.6%	0.000
Exogenous factors	2	83	2.82（0.08，5.55）	49.33	92.6%	0.000

Note: This table presents subgroup analyses of KOOS-SR (Sports and Recreation) outcomes according to stem cell source (autologous vs allogeneic), tissue origin (peripheral blood, adipose-derived, and bone synovial tissue), delivery approach (with vs without scaffold implantation), and disease type (endogenous vs exogenous factors).

Effect estimates are expressed as standardized mean differences (SMD) with 95% confidence intervals (CI). The number of included studies, total sample size, study weights, heterogeneity (I^2), and p values are reported for each subgroup.

All analyses were performed using random-effects models. Within-subgroup heterogeneity was assessed using the I^2 statistic, and reported p values correspond to heterogeneity within each subgroup.

Abbreviations: SMD, standardized mean difference; CI, confidence interval; I^2, heterogeneity statistic; NA, not applicable.

Supplement Table 8. GRADE assessment of certainty of evidence for all outcomes

Outcome	Studies (Participants)	Effect size (SMD, 95% CI)	Risk of bias	Inconsistency	Indirectness	Imprecision	Publication bias	Certainty of evidence
Pain	19 (1008)	-1.31(-1.82, -0.81)	Not serious	Not serious	Not serious	Not serious	Not serious	High
WOMAC	17 (876)	-0.87(-1.09, -0.47)	Not serious	Not serious	Not serious	Not serious	Not serious	High
Cartilage defect score	6 (295)	3.73 (1.69, 5.77)	Not serious	Not serious	Not serious	Serious (small sample / wide CI)	Not serious	Moderate
Cartilage volume	7 (312)	0.91 (0.28, 1.53)	Not serious	Not serious	Not serious	Not serious	Not serious	High
Complications	13 (555)	1.58 (0.72, 3.47)	Not serious	Not serious	Not serious	Not serious	Not serious	High
KOOS-S	4 (371)	3.04 (0.11, 5.96)	Not serious	Serious (heterogeneity)	Not serious	Serious	Not serious	Moderate
KOOS-ADL	6 (418)	2.49 (0.63, 4.36)	Not serious	Serious	Not serious	Serious	Not serious	Moderate
KOOS-SR	4 (371)	2.39 (0.26, 4.51)	Not serious	Serious	Not serious	Serious	Not serious	Moderate
KOOS-QoL	4 (371)	2.21 (0.06, 4.36)	Not serious	Serious	Not serious	Serious	Not serious	Moderate
IKDC	3 (370)	1.90 (-0.11, 3.91)	Not serious	Serious	Not serious	Serious	Not serious	Low

Note：This table summarizes the certainty of evidence for each outcome using the Grading of Recommendations Assessment, Development and Evaluation (GRADE) framework. The assessment includes five domains: risk of bias, inconsistency, indirectness, imprecision, and publication bias.

For each outcome, the number of included studies and participants, pooled effect estimates (standardized mean differences [SMD] with 95% confidence intervals [CI]), domain-specific judgments, and overall certainty of evidence (high, moderate, low, or very low) are reported.

Downgrading of evidence certainty was applied when serious concerns were identified in one or more GRADE domains, particularly due to inconsistency, imprecision, risk of bias, or potential publication bias. The classification reflects an integrated judgment across all domains in accordance with GRADE Working Group recommendations.

Abbreviations: GRADE, Grading of Recommendations Assessment, Development and Evaluation; SMD, standardized mean difference; CI, confidence interval.

Supplement Table 9. Oxford Centre for Evidence-Based Medicine (OCEBM, 2011) Levels of Evidence

Question	Step 1(Level 1*)	Step 2(Level 2*)	Step 3(Level 3*)	Step 4(Level 4*)	Step 5 (Level 5)	Evaluation results
How common is the problem?	Local and current random sample surveys (or censuses)	Systematic review of surveys that allow matching to local circumstances**	Local non-random sample**	Case-series**	n/a	Level 2
Is this diagnostic or monitoring test accurate? (Diagnosis)	Systematic review of cross sectional studies with consistently applied reference standard and blinding	Individual cross sectional studies with consistently applied reference standard and blinding	Non-consecutive studies, or studies without consistently applied reference standards**	Case-control studies, or "poor or non-independent reference standard**	Mechanism-based reasoning	Level 1
What will happen if we do not add a therapy? (Prognosis)	Systematic review of inception cohort studies	Inception cohort studies	Cohort study or control arm of randomized trial*	Case-series or case-control studies, or poor quality prognostic cohort study**	n/a	Level 1
Does this intervention help? (Treatment Benefits)	Systematic review of randomized trials or n-of-1 trials	Randomized trial or observational study with dramatic effect	Non-randomized controlled cohort/follow-up study**	Case-series, case-control studies, or historically controlled studies**	Mechanism-based reasoning	Level 1
What are the COMMON harms? (Treatment Harms)	Systematic review of randomized trials, systematic review of nested case-control studies, n- of-1 trial with the patient you are	Individual randomized trial or (exceptionally) observational study with dramatic effect	Non-randomized controlled cohort/follow-up study (post-marketing surveillance) provided there are sufficient numbers to rule out a common harm. (For	Case-series, case-control, or historically controlled studies**	Mechanism-based reasoning	Level 1

Question	Step 1(Level 1*)	Step 2(Level 2*)	Step 3(Level 3*)	Step 4(Level 4*)	Step 5 (Level 5)	Evaluation results
	raising the question about, or observational study with dramatic effect		long-term harms the duration of follow-up must be sufficient.)**			
What are the RARE harms? (Treatment Harms)	Systematic review of randomized trials or n-of-1 trial	Randomized trial or (exceptionally) observational study with dramatic effect				Level 1
Is this (early detection) test worthwhile? (Screening)	Systematic review of randomized trials	Randomized trial	Non -randomized controlled cohort/follow-up study**	Case-series, case-control, or historically controlled studies**	Mechanism-based reasoning	Level 1

Note：This table presents the classification of evidence levels based on the Oxford Centre for Evidence-Based Medicine (OCEBM) 2011 framework.

Each research question (including prevalence, diagnosis, prognosis, treatment benefits, and harms) is mapped to the corresponding OCEBM evidence hierarchy (Levels 1–5), which reflects the strength of evidence according to study design, methodological rigor, and consistency of findings.

Level 1 represents the highest level of evidence (e.g., systematic reviews of randomized controlled trials or high-quality RCTs), while Level 5 represents expert opinion or mechanism-based reasoning. The final evidence level for each domain is reported in the “Evaluation results” column.

Abbreviations: OCEBM, Oxford Centre for Evidence-Based Medicine.
